# RNF43 p.G659fs leads to natural killer cell dysfunction in MSI-high colorectal cancer through PI3K/AKT/mTOR signaling and HLA-E up-regulation

**DOI:** 10.1126/sciadv.adz6907

**Published:** 2026-09-23

**Authors:** Pushpamali De Silva, Samantha Fitzgerald, Jules Cazaubiel, Kristin Qian, Matan Hofree, Jia-Ren Lin, Roxanne Pelletier, Mariana Lopez Leon, Phuong Vu Mai Ho, Yasutoshi Takashima, Juha P Väyrynen, Kosuke Matsuda, Tomotaka Ugai, Dane Ford-Roshon, Pratyusha Bala, Nir Hacohen, Kimmie Ng, Jonathan A. Nowak, Shuji Ogino, Peter K. Sorger, Marios Giannakis

**Affiliations:** ^1^ Department of Medical Oncology, Dana-Farber Cancer Institute, Harvard Medical School, Boston, MA, USA.; ^2^ Broad Institute of MIT and Harvard, Cambridge, MA, USA.; ^3^ Laboratory of Systems Pharmacology, Harvard Medical School, Boston, MA, USA.; ^4^ Translational Medicine Research Unit, Medical Research Center Oulu, Oulu University Hospital, and University of Oulu, Oulu, Finland.; ^5^ Department of Pathology, Brigham and Women’s Hospital and Harvard Medical School, Boston, MA, USA.; ^6^ Department of Epidemiology, Harvard T.H. Chan School of Public Health, Boston, MA, USA.; ^7^ Division of Integrative Cancer Research, National Cancer Center Research Institute, Tokyo, Japan.; ^8^ Harvard Medical School, Boston, MA, USA.; ^9^ Department of Medicine, Massachusetts General Hospital Cancer Center, Boston, MA, USA.; ^10^ Cancer Immunology and Cancer Epidemiology Programs, Dana-Farber Harvard Cancer Center, Boston, MA, USA.; ^11^ Institute of Science Tokyo, Tokyo, Japan.

## Abstract

The RNF43 p.G659fs (*RNF43^659mut^*) mutation occurs in 8% of colorectal cancers (CRCs) and is enriched in microsatellite instability–high (MSI-H) tumors. Although *RNF43^659mut^* promotes tumor growth through phosphatidylinositol 3-kinase/protein kinase B/mechanistic target of rapamycin (PI3K/AKT/mTOR) activation independent of WNT signaling, its immunologic effects are poorly defined. We show that *RNF43^659mut^* shapes an immunosuppressive natural killer (NK) cell landscape in MSI-H CRC. Single-cell RNA sequencing and multiplex immunohistochemistry revealed increased NK cell infiltration in *RNF43^659mut^* tumors, yet these cells displayed inhibitory phenotypes. Bulk RNA sequencing and flow cytometry of NK cells cocultured with *RNF43^659mut^* isogenic lines and MSI-H patient-derived organoids demonstrated reduced functional NK subsets, decreased activation markers, increased inhibitory receptors, and impaired cytotoxicity. Mechanistically, *RNF43^659mut^*-mediated PI3K/AKT activation induced HLA-E expression, suppressing NK cell function. Pharmacological or small interfering RNA–mediated PI3K/AKT inhibition restored NK cell activity and enhanced tumor cell killing. Spatial analysis of MSI-H patient tumors revealed close proximity between NKG2A^+^NK cells and HLA-E^+^ tumor cells, which were themselves adjacent to PI3K/AKT-activated tumor cells, highlighting a localized immunosuppressive niche. These findings uncover an immune evasion mechanism in MSI-H CRC, implicating the PI3K/AKT-HLA-E/NKG2A axis as a promising therapeutic target to overcome immunotherapy resistance.

## INTRODUCTION

Colorectal cancer (CRC) remains a significant global health challenge, consistently ranking among the leading causes of cancer-related deaths worldwide (*1*, *2*). Despite advances in screening, early detection, and treatment, CRC continues to have high morbidity and mortality rates and an alarming rise in early-onset CRC, defined as diagnoses in individuals under the age of 50 (*3*). CRC exhibits molecular heterogeneity and treatment outcomes can often be predicted on the basis of the tumor’s molecular profile (*4*–*6*). Microsatellite instability–high (MSI-H) tumors, caused by defects in the DNA mismatch repair (MMR) system, typically generate numerous neoantigens. These neoantigens enhance the tumor’s visibility to the immune system, making MSI-H CRCs more responsive to immunotherapy compared to microsatellite-stable (MSS) CRCs. However, despite this immunogenic potential, not all patients with MSI-H CRC experience favorable responses to immunotherapy, highlighting the presence of underlying mechanisms of immune evasion that compromise treatment efficacy. Unraveling these mechanisms is crucial to optimizing therapeutic strategies and ensuring better outcomes for patients with CRC.

*RNF43* was identified as a frequently mutated gene in CRC (*7*). *RNF43* encodes an E3 ubiquitin ligase that, in its intact form, functions as a negative regulator of the Wnt signaling pathway, which is crucial for cellular proliferation, differentiation, and survival (*8*, *9*). Mutations in *RNF43* have been identified in ∼15 to 20% of CRCs (*7*, *10*, *11*). Among these, the most frequently observed mutation is the C-terminal domain alteration, *RNF43 p.G659fs* (*RNF43^659mut^*), present in around 8% of all CRCs and enriched in MSI-H tumors, where it occurs in ∼22 to 42% of cases (*7*, *12*, *13*). Unlike inactivating, loss-of-function *RNF43* mutations, the *RNF43^659mut^* mutation disrupts the normal function of RNF43, leading to aberrant activation of the phosphatidylinositol 3-kinase (PI3K)/protein kinase B (AKT)/mechanistic target of rapamycin (mTOR) signaling in CRC cells and drives tumor growth independently of the Wnt signaling pathway (*14*). The activation of the PI3K/AKT pathway in cancer cells is well recognized for its profound impact on immune cell function and contributes to immune evasion within the tumor microenvironment (TME) (*15*–*18*). However, the specific consequences of aberrant PI3K/AKT activation via *RNF43^659mut^* on the immune microenvironment in MSI-H CRC remain unknown.

To understand better the immune landscape and immune cell alterations in *RNF43^659mut^* MSI-H CRC in an unbiased way, we used single-cell RNA sequencing (scRNA-seq) of primary tumors. We validated and integrated results with multiple experimental approaches, including MSI-H CRC patient tumor analyses using multiplex immunofluorescence (mIF), cyclic immunofluorescence (CyCIF) (*19*), and in vitro tumor–natural killer (NK) coculture assays with isogenic tumor lines and patient-derived organoids (PDOs). This research could not only provide valuable insights into the mechanisms of immune evasion in MSI-H CRC and identify potential therapeutic strategies to overcome resistance to immunotherapy in MSI-H CRCs (*20*) but also have broader implications for CRC immunomodulation.

## RESULTS

### Differential immune profiles in *RNF43^659mut^* versus *RNF43^wt^* MSI-H CRC tumors

**Fig. 1.**
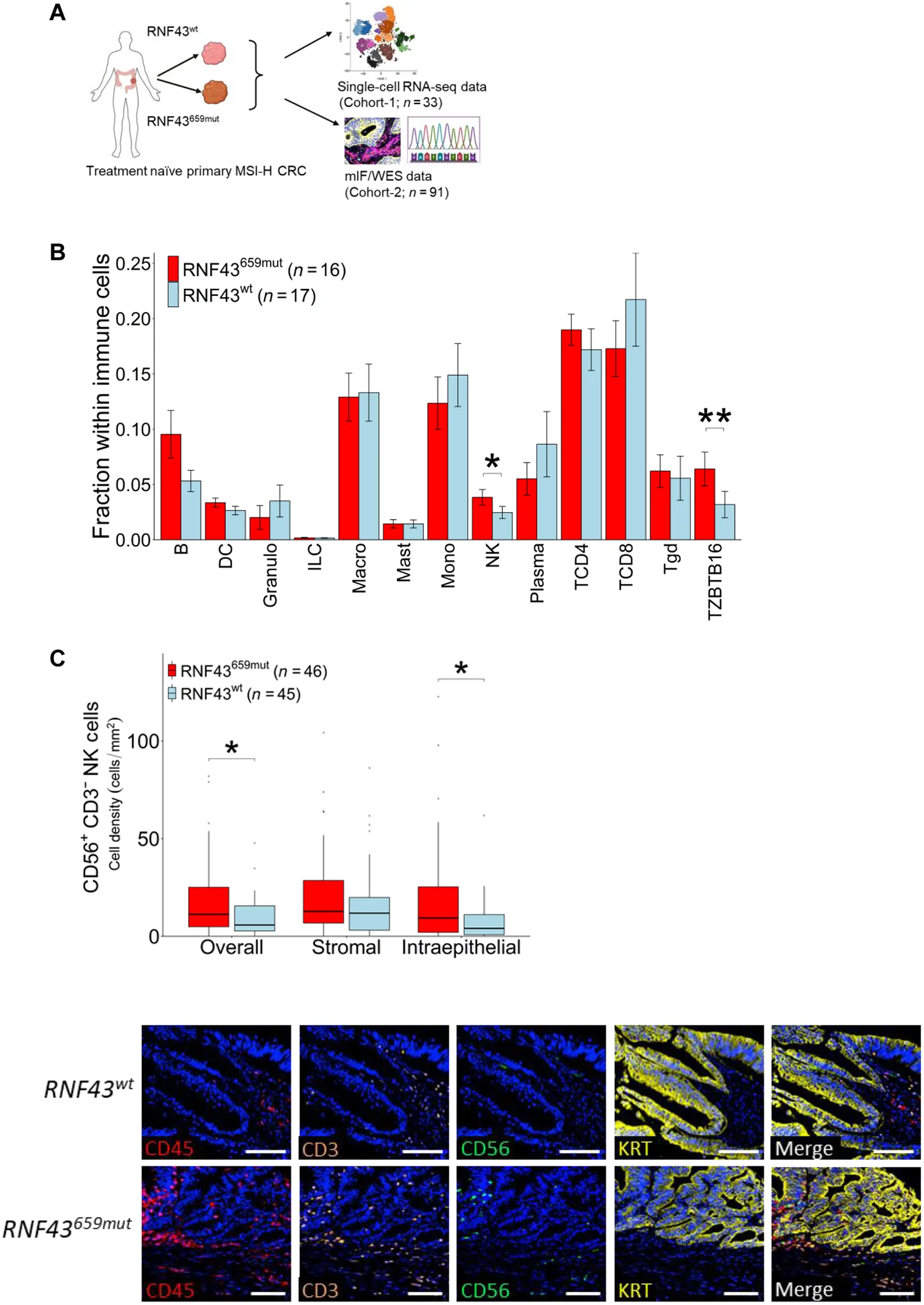
Differential immune profiles in *RNF43^659mut^* versus *RNF43^wt^* MSI-H CRC tumors. (**A**) Schematic overview of two independent MSI-H CRC patient cohorts analyzed to assess the impact of *RNF43^659mut^* on immune cell profiles in the TME. Cohort-1 (*n* = 33) underwent scRNA-seq, whereas Cohort-2 (*n* = 91) was analyzed using mIF. Created in BioRender. De Silva, P. (2026) https://BioRender.com/7c7khiv. (**B**) Immune cell deconvolution of scRNA-seq data from Cohort-1. (**C**) Top: NK cell densities in Cohort-2 using mIF in *RNF43^659mut^* tumors relative to *RNF43^wt^* tumors. Overall: NK cell density in intraepithelial plus stromal compartments combined. Bottom: Representative mIF images to show CD3^−^CD56^+^ NK cells in *RNF43^wt^* and *RNF43^659mut^* tumors. CD45, pan-leukocytes; CD3, pan-T cells; CD56, NK cells; KRT, keratin. KRT channel is reused in fig. S1C for orientation and comparison across figures. For (B) and (C), significance was determined by the Mann-Whitney *U* test. Asterisks denote statistical significance (*P < 0.05). Scale bars, 100 μm.

To investigate the impact of *RNF43^659mut^* on tumor immune landscape, we analyzed two independent MSI-H CRC patient cohorts (Fig. 1A). We used scRNA-seq data from untreated primary MSI-H CRCs (Cohort-1; *n* = 33) to identify immune cell differences between *RNF43^659mut^* and *RNF43^wt^* CRC. We found that NK cells and a rare population of T cells expressing the ZBTB16 transcription factor were significantly enriched in the TME of *RNF43^659mut^* tumors compared to *RNF43^wt^* tumors (Fig. 1B), whereas other immune cell subsets, including B cells, other T cell subsets, and myeloid cell populations, showed no significant differences in abundance between the two groups. We also analyzed a second cohort (Cohort-2; *n* = 91) of MSI-H primary CRCs with mIF spatial immune cell data (*21*) to validate the scRNA-seq findings. Consistent with Cohort-1, *RNF43^659mut^* tumors showed a significantly higher density of CD56^+^CD3^−^ NK cells in overall tumor areas (intraepithelial plus stromal areas) and in intraepithelial areas compared to *RNF43^wt^* tumors (Fig. 1C). In addition, *RNF43^659mut^* tumor cells had a higher tumor mutational burden (TMB) and decreased β2-microglobulin (*B2M*) expression (fig. S1, A to C). Despite the lower *B2M* expression in *RNF43^659mut^* tumor cells, classical HLA-I (HLA-A, HLA-B, and HLA-C) expression remained relatively unchanged (fig. S1B), suggesting that NK cell infiltration may be influenced by alternative mechanisms in addition to a “missing self” response.

### Increased expression of NK cell inhibitory receptors and decrease in activation markers in *RNF43^659mut^*-NK cell cocultures

**Fig. 2.**
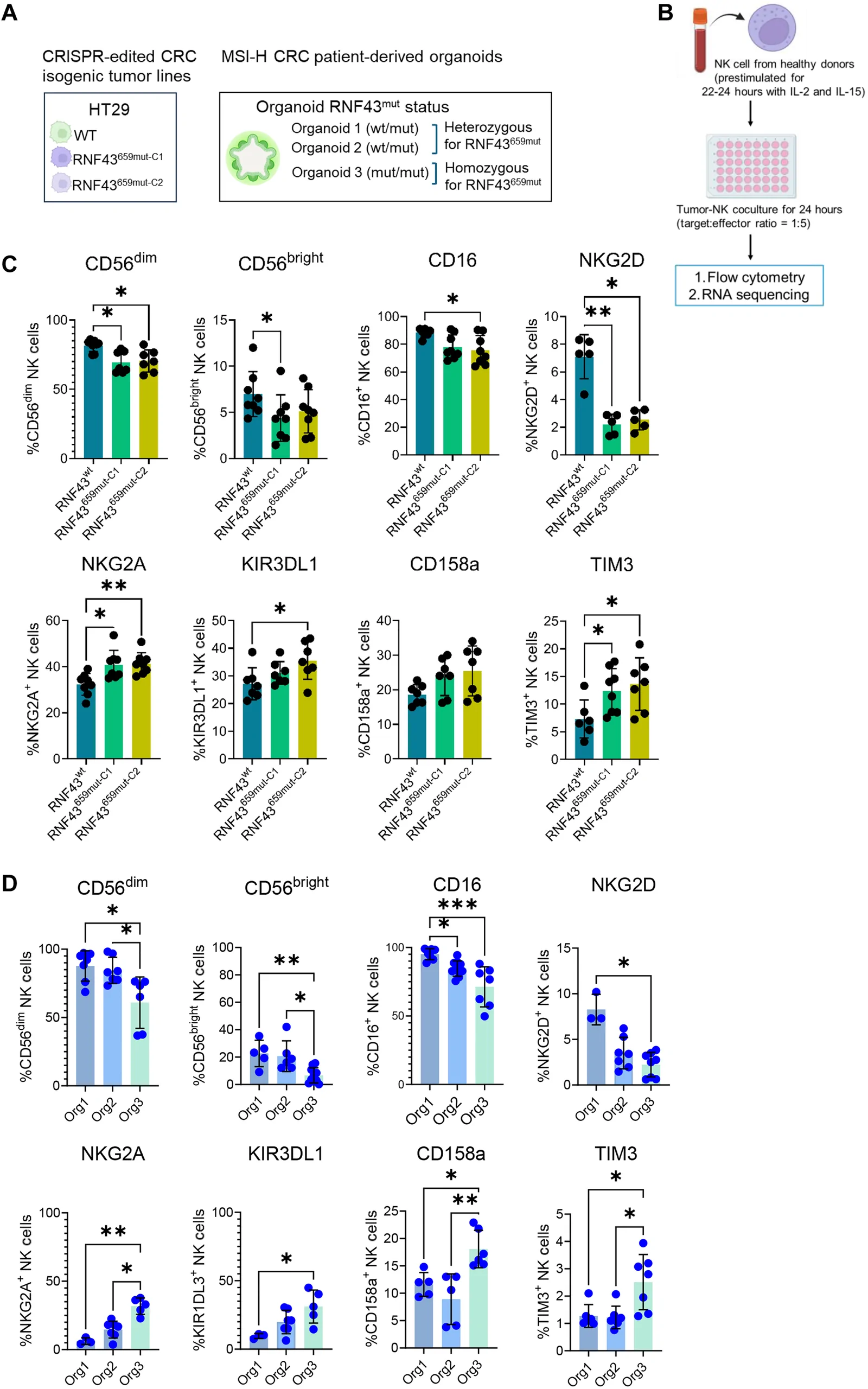
*RNF43^659mut^* drives NK cell dysfunction through enhanced inhibitory and decreased activation signaling. (**A**) Schematic depiction of the isogenic HT29 tumor lines and PDO models used in NK cell tumor coculture assays. The HT29 isogenic lines include *RNF43^wt^* (wild type) and two mutant single clones: RNF43^659mut_C1^ and RNF43^659mut_C2^. Organoid models (Org1: wt/mut, Org2: wt/mut, and Org3: mut/mut) display varying allelic frequencies, reflecting tumor heterogeneity. (**B**) Workflow illustrating the isolation, stimulation, and preparation of healthy donor peripheral blood–derived NK cells for coculture with tumor isogenic lines and organoid models. Multicolor flow cytometry analysis of the NK cell lineage (CD56^dim^, CD56^bright^, and CD16), activation (NKG2D), and inhibitory markers (NKG2A, KIR3DL1, CD158a, and TIM3) in cocultures of (**C**) HT29 isogenic tumor lines and (**D**) PDOs. Data in (C) and (D) are presented as mean ± SD of pooled >3 independent biological experiments done in duplicates. Each point denotes NK cells from a different healthy donor. Significance was determined by a Kruskal-Wallis with Dunn’s multiple-comparisons test. Asterisks denote statistical significance (*P < 0.05; **P < 0.01; ***P < 0.001).

As our analyses showed enrichment of NK cells in *RNF43^659mut^* CRCs, we sought to understand how this mutation may affect NK cell function. To that end, we performed in vitro tumor-NK cell coculture assays using CRISPR-edited isogenic CRC cell lines, MSI-H CRC PDOs, and healthy donor-derived NK cells (Fig. 2A). The isogenic CRC models include HT29 wild-type (wt) and two HT29 single clones of *RNF43^659mut^* (-C1 and -C2), whereas the organoid (Org) models consist of Org1 and Org2 that are heterozygous for *RNF43^659mut^* (*RNF43^wt^*/*RNF43^659mut^*: wt/mut) and Org3, which is homozygous (*RNF43^659mut^*/*RNF43^659mut^*: mut/mut). This selection allowed for a comprehensive assessment of NK cell activity across both engineered and patient-derived tumor models. Prestimulated healthy donor NK cells cocultured with *RNF43^659mut^* HT29 tumor cells over 24 hours (Fig. 2B) exhibited significant down-regulation of CD56^dim^ and CD56^bright^ NK cell subsets and activation markers including CD16 and NKG2D in *RNF43^659mut^* compared to wild-type isogenic controls (Fig. 2C). We noted up-regulation of inhibitory markers such as NKG2A, TIM-3, and KIR3DL1 in *RNF43^659mut^*. We also recapitulated these results in NK cells cocultured with PDOs where we observed a consistent pattern of decreased expression of activation markers and increased expression of inhibitory receptors in the homozygous (mut/mut) organoid model (Org3) compared to heterozygous (mut/wt) PDOs (Fig. 2D). Supporting these findings, analysis of scRNA-seq data reveal distinct transcriptional profiles in NK cells derived from Cohort-1 tumors, highlighting increased expression of genes associated with immune exhaustion (fig. S2A). In addition, RNA-seq of fluorescence-activated cell sorting (FACS)–sorted NK cells cocultured with HT29 isogenic tumor lines was consistent with the flow cytometry data, confirming a dysregulated immune phenotype characterized by impaired NK cell activation and heightened inhibitory signaling (fig. S2B). Specifically, NK cells sorted from *RNF43^659mut^* cocultures exhibited decreased expression of activation- and cytotoxicity-associated genes (e.g., *NCR3*, *TNFRSF9*, *CD83*, and *IL2RA*) together with increased expression of inhibitory and exhaustion-associated genes (e.g., *HAVCR2*, *KLRC1*, and *CD274*), supporting a tumor cell–intrinsic effect of *RNF43^659mut^* on NK cell dysfunction. Together, these results demonstrate that *RNF43^659mut^* in CRC cells may induce a suppressive immune microenvironment to impair NK cell activity through increased inhibitory signaling and decreased activation. The reduced expression of NK lineage and activation markers observed in coculture reflects functional impairment of NK cells rather than reduced NK cell abundance, consistent with the increased NK cell infiltration observed in patient tumors (Fig. 1).

### *RNF43^659mut^* impairs NK cell cytotoxicity in CRC

**Fig. 3.**
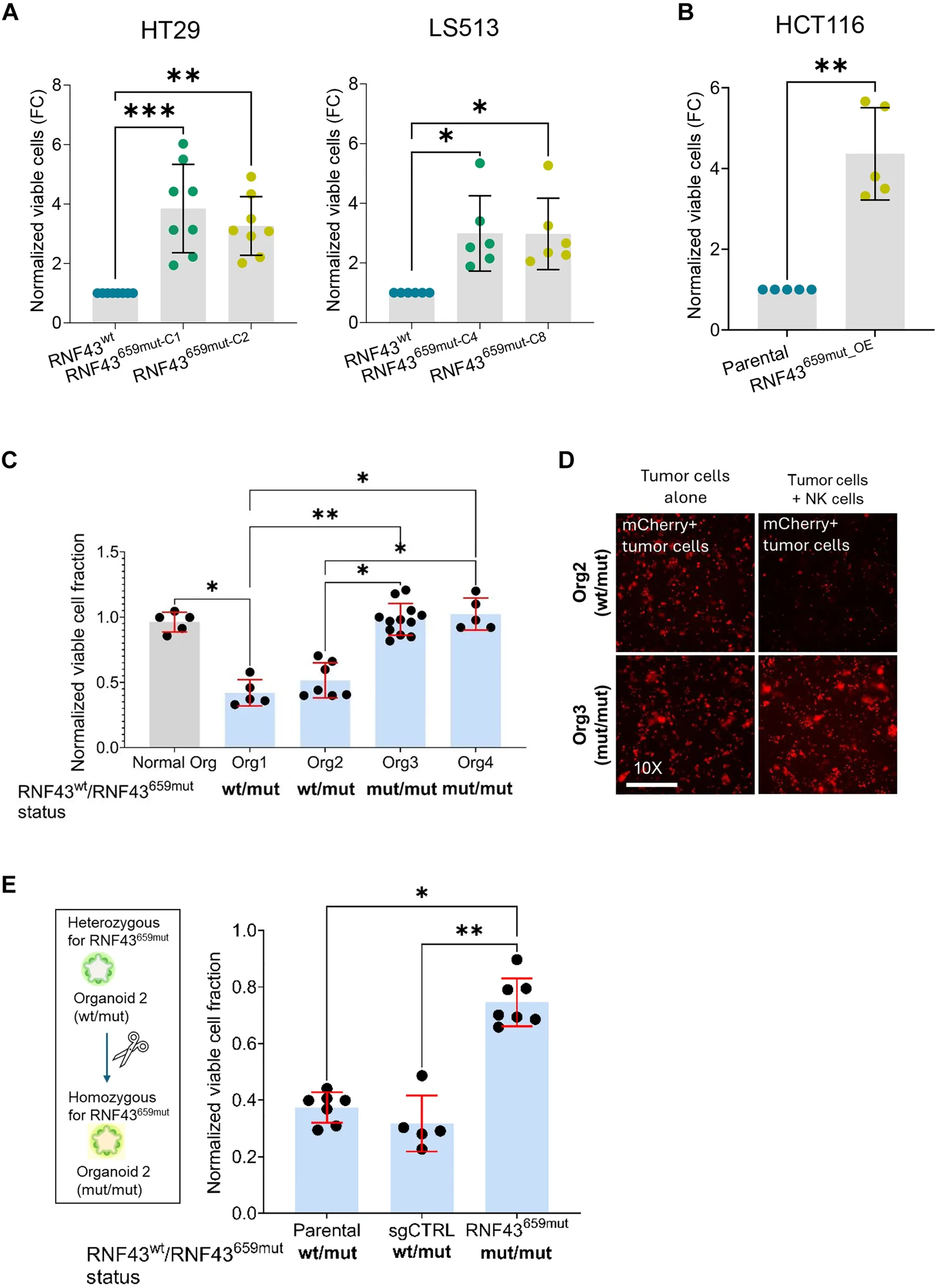
*RNF43^659mut^* impairs NK cell cytotoxicity across CRC tumor models. Assessment of NK cell–mediated cytotoxicity in cocultures with (**A**) CRISPR-edited isogenic HT29 and LS513 CRC tumor lines. Tumor cell viability was measured using flow cytometry and is shown as a fold change (FC) comparing *RNF43^659mut^* lines with *RNF43^wt^* controls. The LS513 isogenic mutant clones are RNF43^659mut-C4^ and RNF43^659mut-C8^. (**B**) *RNF43^659mut_OE^* MSI-H HCT116 CRC tumor line. Tumor cell viability was measured using flow cytometry and is shown as an FC comparing *RNF43^659mut_OE^* to the parental line. (**C**) MSI-H PDOs. Organoid viability was measured using flow cytometry and is shown as the viable cell fraction normalized to PDOs cultures without NK cells (monoculture). The normal organoid line was used as a control for NK cell killing. (**D**) Representative live-cell images of mCherry-expressing Org2 and Org3 organoids cocultured with NK cells for 24 hours. Magnification: 10x. (**E**) CRISPR-edited MSI-H CRC PDOs. Left: Schematic of the conversion of heterozygous *RNF43^659mut^* PDOs to homozygous *RNF43^659mut^.* Right: Organoid viability was measured using flow cytometry in parental, sgCTRL, and *RNF43^659mut^* edited Org2 cocultured with NK cells after 24 hours. In (A), (B), (C), and (E), data are presented as mean ± SD of pooled >3 independent biological experiments done in duplicates. Each point denotes NK cells from a different healthy donor. NK cell killing in NK-tumor cocultures was first normalized to viability in tumor monocultures before assessing FC or viable cell fraction. Statistical significance was determined using a Kruskal-Wallis with Dunn’s multiple-comparisons test for (A), (C), and (E) and a Mann-Whitney *U* test for (B). Asterisks denote statistical significance (*P < 0.05; **P < 0.01; ***P < 0.001).

Given the observed increase in inhibitory and decrease in activation NK cells markers in cocultures, we hypothesized that *RNF43^659mut^* would impair NK cell–mediated cytotoxicity in CRC. We found a significant reduction in NK cell–mediated cytotoxicity toward *RNF43^659mut^* HT29 and LS513 isogenic CRC tumor lines (Fig. 3A). NK cell cytotoxicity in tumor-NK cocultures were analyzed by flow cytometry; representative plots and the gating strategy are shown in fig. S3. NK cell–mediated cytotoxicity was also evaluated in cocultures with an *RNF43^659mut^*-overexpressing (*RNF43^659mut_OE^*) MSI-H CRC cell line (HCT116), which displayed significantly reduced NK cell killing compared to the parental HCT116 line (Fig. 3B). In addition, MSI-H CRC PDOs homozygous (mut/mut) for *RNF43^659mut^* displayed resistance to NK cell–mediated cytotoxicity compared to heterozygous (wt/mut) PDOs (Fig. 3C). We also used a normal organoid line, established from an adjacent normal tissue specimen, as a control for the NK cytotoxic assay and observed limited NK cell–mediated killing (Fig. 3C). This observed resistance in the normal organoid reflects normal self-tolerance rather than tumor-intrinsic immune evasion and is consistent with physiological self-protection mechanisms in normal epithelial cells, which engage inhibitory pathways to maintain NK cell tolerance (*22*–*24*). This allele dose–dependent resistance phenotype was further confirmed using mCherry-labeled PDOs, where NK cell coculture resulted in pronounced loss of mCherry signal in wt/mut organoid 2 but robust preservation of viable tumor cells in mut/mut organoid 3 (Fig. 3D). Moreover, we were able to confer this resistance with CRISPR editing of heterozygous *RNF43^659mut^* PDOs (organoid 2) to homozygosity for *RNF43^659mut^* (Fig. 3E). The dose-dependent effect of *RNF43^659mut^* allelic burden in coculture on organoid viability shows a correlation in PDOs between increased mutant allele frequency and heightened resistance to NK cell–mediated cytotoxicity. Collectively, these findings demonstrate that *RNF43^659mut^* confers resistance to NK cell–mediated cytotoxicity across various CRC models.

### PI3K/AKT/mTOR pathway activation with *RNF43^659mut^* leads to NK cell immune evasion via HLA-E up-regulation

**Fig. 4.**
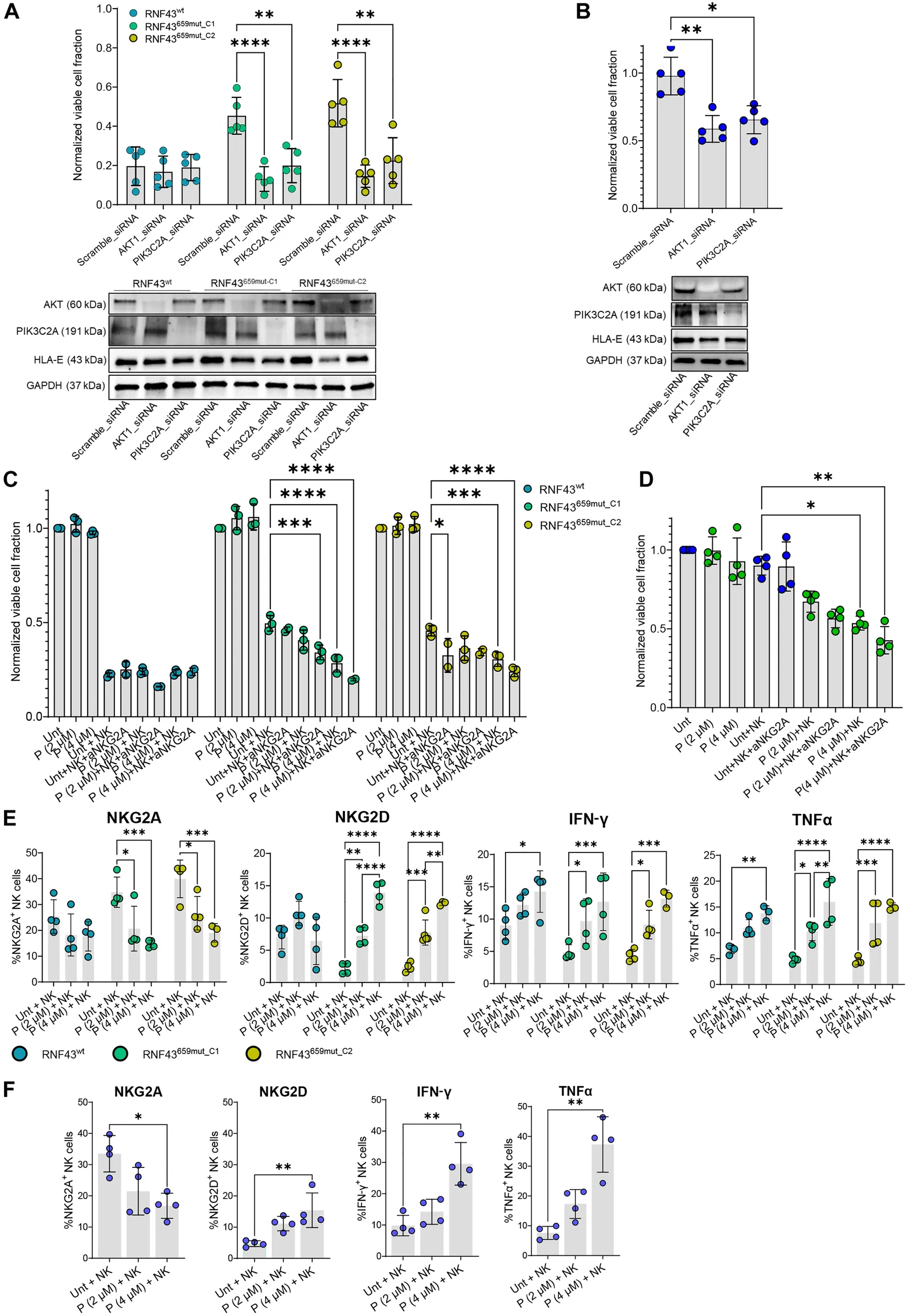
PI3K/AKT pathway inhibition restores NK cell cytotoxicity by modulating HLA-E expression. Knockdown of the PI3K/AKT pathway using siRNA targeting *AKT1* and *PIK3C2A* and tumor cell viability (top) and AKT, PIK3C2A, and HLA-E expression (bottom) in (**A**) *RNF43^659mut^* HT29 isogenic lines and (**B**) *RNF43^659mut^* homozygous PDOs (Org3) cocultured with NK cells. Viability was normalized to scramble siRNA controls. Pharmacological inhibition of the PI3K/AKT pathway using sublethal doses of perifosine (P: 2 and 4 μM) in *RNF43^659mut^* (**C**) HT29 isogenic lines and (**D**) Org3 cocultured with NK cells. The normalized viable cell fraction was determined under the conditions: Unt: untreated tumor cells; P (2 μM)/P (4 μM): tumor cells treated with perifosine at 2 or 4 μM alone; Unt + NK: untreated tumor cells cocultured with NK cells; P (2 μM) + NK/P (4 μM) + NK: tumor cells treated with perifosine at 2/4 μM and then cocultured with NK cells; Unt + NK + α-NKG2A: untreated tumor cells cocultured with NK cells in the presence of monalizumab (anti-NKG2A blocking antibody); P (2 μM) + NK + α-NKG2A/P (4 μM) + NK + α-NKG2A: tumor cells treated with perifosine at 2/4 μM and then cocultured with NK cells in the presence of monalizumab. NKG2A, NKG2D, IFN-γ, and TNFα expression in NK cells cocultured with perifosine-treated *RNF43^659mut^* (**E**) HT29 isogenic lines and (**F**) Org3. In (A) to (E), data are presented as mean ± SD of pooled ≥3 independent biological experiments done in duplicates. Each point denotes NK cells from a different healthy donor. NK killing in NK-tumor cocultures was first normalized to viability in tumor monocultures before assessing viable cell fraction using flow cytometry. Statistical significance was determined using a Kruskal-Wallis with Dunn’s multiple-comparisons test. Asterisks denote statistical significance (*P < 0.05; **P < 0.01; ***P < 0.001, ****P < 0.0001).

We postulated that the aberrant activation of PI3K/AKT/mTOR signaling with *RNF43^659mut^* (*14*) could lead to NK cell dysfunction due to the well-established role of this pathway in immune evasion (*15*–*18*). We observed evidence consistent with enhanced PI3K/AKT/mTOR pathway activity in *RNF43^659mut^* across multiple MSI-H CRC models, including epithelial cells profiled by scRNA-seq in Cohort-1, bulk RNA-seq of HT29 isogenic lines, and immunoblot analyses in HT29 isogenic lines, *RNF43^659mut_OE^* HCT116 cells, PDOs, and CRISPR-edited organoids (Fig. 4, A and B, and fig. S4, A to C). Epithelial cell scRNA-seq analysis in patient Cohort-1 revealed transcriptional shifts in *RNF43^659mut^* compared to *RNF43^wt^* tumors, characterized by the up-regulation of PI3K/AKT/mTOR-regulating genes such as *HOX* genes (*25*), *SOX2* (*26*), and *CD109* (*27*), indicating altered cellular states, including enhanced epithelial-mesenchymal transition (EMT), increased proliferative capacity, and reduced immune responsiveness, all of which contribute to tumor progression (fig. S4A). Bulk RNA-seq of HT29 isogenic tumor cells demonstrated enrichment of transcriptional programs associated with PI3K/AKT/mTOR pathway activity and may also contribute to immune evasion. Specifically, genes involved in AKT/mTOR signaling and downstream translational regulation, including *FOXO1*, *FOXO3*, *EIF4EBP1*, and *RICTOR*, exhibited expression patterns consistent with increased pathway activity in *RNF43^659mut^* compared with *RNF43^wt^* lines. In addition, we observed increased expression of genes implicated in inflammatory and immune regulatory signaling, including *TNFRSF1B*, *NFKB1*, *NFKB2*, *USP18*, *IDO1*, and *TGF-β1* (*28*–*30*) (fig. S4B).

In parallel, *RNF43^659mut^* cells exhibited increased expression of immune checkpoint and inhibitory regulators such as CD274 [programmed death molecule ligand 1 (PD-L1)] and LGALS9, along with coordinated induction of interferon-stimulated genes including MX1, OAS2, IFIT1, RSAD2, and DDX60, consistent with chronic inflammatory and interferon-associated signaling states that can promote immune resistance and tumor progression. Notably, HLA-E, an inhibitory ligand that suppresses NK cell–mediated cytotoxicity, was markedly up-regulated in *RNF43^659mut^* isogenic cells compared with *RNF43^wt^* controls, supporting its role in facilitating NK cell immune evasion (fig. S4B). Immunoblot analysis and quantification of HLA-E expression further supported increased HLA-E levels across multiple cell line and organoid models concomitant with PI3K pathway activity. Specifically, *RNF43^659mut^* was associated with significantly elevated HLA-E expression in HT29 isogenic tumor lines, *RNF43^659mut_OE^* HCT116 cells, and homozygous CRC PDOs, including Org2 (wt/mut) following CRISPR-mediated editing to homozygosity (fig. S4C).

Together, these findings, supported by transcriptional and protein-level analyses, indicate enhanced PI3K/AKT/mTOR signaling in *RNF43^659mut^* models associated with increased HLA-E expression and resistance to NK cell–mediated cytotoxicity. These observations support a dual role for *RNF43^659mut^* in promoting both tumor progression and immune evasion in MSI-H CRC.

### PI3K/AKT pathway inhibition restores NK cell function by modulating HLA-E expression

We next evaluated the potential for PI3K/AKT pathway inhibition to restore NK cell–mediated cytotoxicity in *RNF43^659mut^* CRC. Small interfering RNA (siRNA) knockdown of *AKT1* and *PIK3C2A* in *RNF43^659mut^* HT29 isogenic lines and Org3 cocultured with NK cells significantly reduced tumor cell viability (Fig. 4, A and B, top) to similar levels as that with *RNF43^wt^* cocultures (Fig. 4A). This restoration of NK cell cytotoxicity was mediated by PI3K/AKT pathway inhibition and reduction in HLA-E expression (Fig. 4, A and B, bottom; immunoblotting data quantified in fig. S5, A and B). Pharmacological inhibition of the PI3K/AKT pathway using perifosine, an AKT inhibitor that prevents AKT membrane localization and phosphorylation (*31*, *32*), also showed a dose-dependent restoration of NK cell–mediated killing in NK cell–tumor cocultures (Fig. 4, C and D, and fig. S5, C and D). This was mediated by PI3K/AKT signaling inhibition and HLA-E down-regulation, which was more pronounced at the time of NK cell cytotoxicity assessment, 24 hours post–drug removal (fig. S5, E and F). Although PI3K/AKT pathway inhibition with siRNA and perifosine did not completely abolish AKT phosphorylation, this partial suppression was sufficient to reduce HLA-E expression and restore NK cell cytotoxicity. These findings suggest that full pathway inhibition may not be required to reverse immune suppression through modulation of the PI3K/AKT-HLA-E axis. Moreover, in contrast to the reduced viability observed with continuous perifosine exposure in monoculture (fig. S5C), short-term treatment with sublethal doses of perifosine (Fig. 4C) minimizes direct drug toxicity, such that the observed effects primarily reflect NK cell–mediated cytotoxicity. To further evaluate therapeutic strategies targeting NK cell suppression, we next examined whether PI3K/AKT inhibition could be combined with NK cell immunotherapy using the anti-NKG2A monoclonal antibody (mAb) monalizumab. Combination treatment with perifosine and monalizumab further enhanced NK cell–mediated cytotoxicity in HT29 *RNF43^659mut^* isogenic lines and *RNF43^659mut^* homozygous PDOs compared with either treatment alone (Fig. 4, C and D). We further found dose-dependent down-regulation of NKG2A on NK cells, coupled with up-regulation of NK cell activation markers, including NKG2D, interferon-γ (IFN-γ), and tumor necrosis factor–α (TNFα) with PI3K/AKT inhibition (Fig. 4, E and F), consistent with the enhanced NK cell–mediated cytotoxicity observed.

To directly determine whether HLA-E itself mediates NK cell suppression in *RNF43^659mut^* CRC, we generated CRISPR-mediated *HLA-E* knockout tumor lines in the *RNF43^659mut^* background and evaluated NK cell cytotoxicity in coculture. HLA-E loss resulted in a partial but significant restoration of NK cell–mediated killing in both the HT29 *RNF43^659mut_C2^* isogenic line and organoid 3, demonstrating that HLA-E contributes directly to immune evasion in *RNF43^659mut^* models (fig. S6, A and B). Notably, the magnitude of cytotoxicity rescue was less pronounced than that achieved via PI3K/AKT pathway inhibition, which not only reduces HLA-E expression but may also have modulated additional immunosuppressive pathways downstream of PI3K/AKT. These findings support a model in which *RNF43^659mut^*-driven PI3K/AKT activation promotes NK cell dysfunction primarily through HLA-E up-regulation while also engaging broader suppressive mechanisms that collectively reinforce immune escape.

Trogocytosis has been reported to induce NK cell dysfunction as NK cells can acquire suppressive ligands through the transfer of tumor cell membrane fragments (*33*–*35*). To determine whether trogocytosis contributes to NK dysfunction, we performed time-course coculture assays (0 to 24 hours) with HT29 isogenic lines and organoid 3, analyzed by flow cytometry, as shown in representative plots (fig. S7, A and B). NK cells displayed transient and limited HLA-E acquisition (<20%), peaking at early time points (1 to 4 hours) and declining by 24 hours (fig. S8C). This pattern is consistent with membrane transfer rather than de novo induction and reflects the already elevated HLA-E on *RNF43^659mut^* tumor cells due to PI3K/AKT activation. These findings indicate that HLA-E trogocytosis can occur but likely represents a minor, ancillary event, whereas tumor-intrinsic HLA-E up-regulation remains the dominant driver of NK suppression in *RNF43^659mut^* CRC. Although a previous study has implicated HLA-G–mediated trogocytosis in NK dysfunction (*33*), we did not detect HLA-G protein expression in the mutant tumor lines examined (fig. S8).

### Spatial analysis of *RNF43^659mut^* tumors reveals PI3K/AKT-linked immune checkpoint evasion via HLA-E/NKG2A

**Fig. 5.**
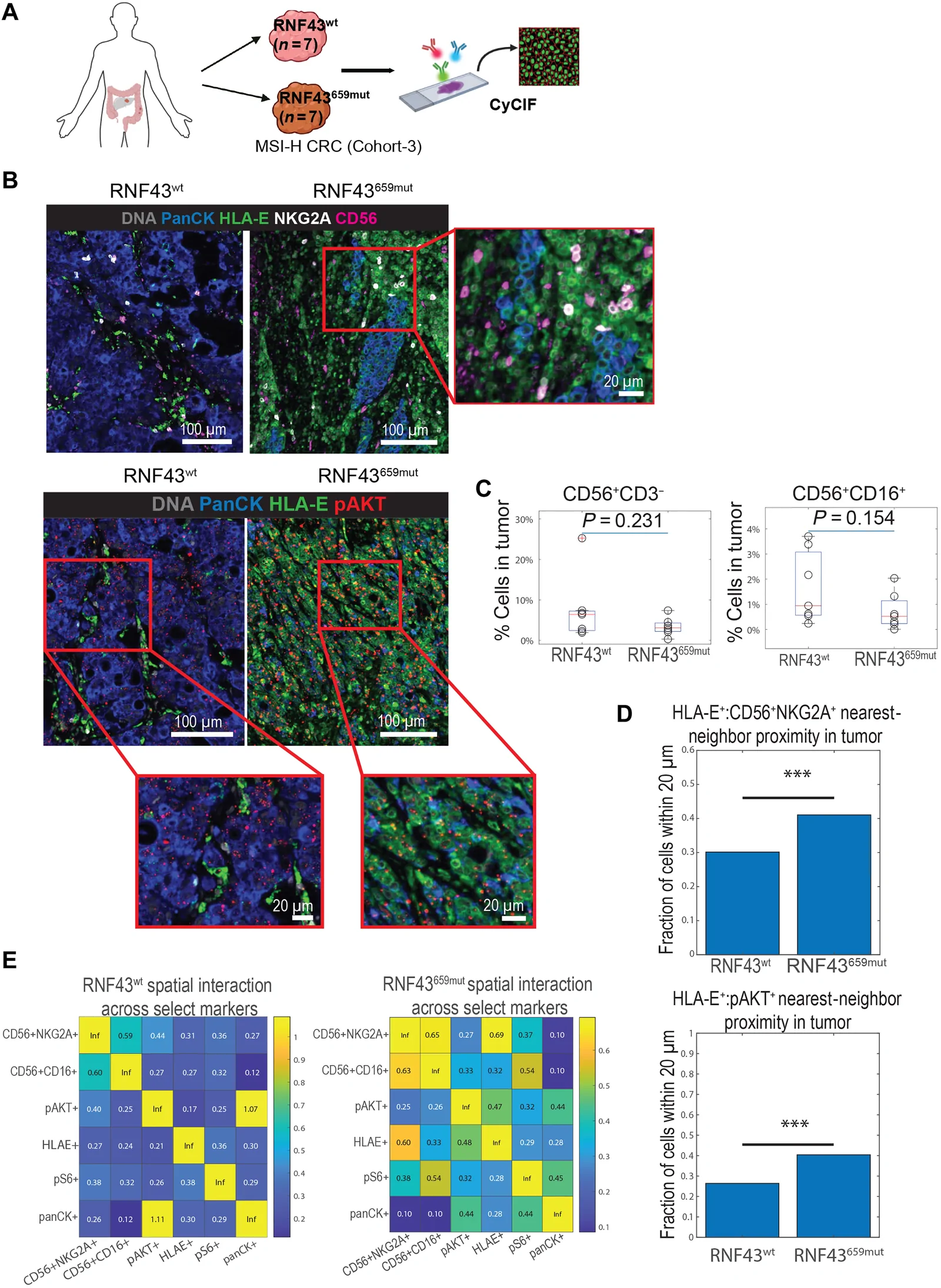
Spatial analysis of the TME in *RNF43^659mut^* versus *RNF43^wt^* CRC. (**A**) Overview of the CyCIF multiplexed single-cell imaging strategy used to analyze the TME, including tumor and NK cell interactions, in *RNF43^659mut^* (*n* = 7) versus *RNF43^wt^* (*n* = 7) MSI-H CRC tumors (Cohort-3). Created in BioRender. De Silva, P. (2026) https://BioRender.com/wh94d2t. (**B**) Top: Representative CyCIF images of CD56^+^NKG2A^+^ NK cell infiltration and location in *RNF43^659mut^* compared to *RNF43^wt^* tumors. Bottom: Representative CyCIF images of HLA-E expression and PI3K/AKT pathway activation (pAKT) in tumor epithelial cells. Scale bars, 100 and 20 μm (insets). PanCK (pan Cytokeratin): tumor epithelial cells. (**C**) Proportions of CD56^+^CD3^−^ NK cells and CD56^+^CD16^+^ NK cells in the tumor region in *RNF43^wt^* and *RNF43^659mut^* samples; *P* values calculated using the Welch’s *t* test. (**D**) Local proximity between cell populations in tumor regions was quantified by measuring the fraction of cells whose nearest neighbor lies within a fixed 20-μm radius using a two-nearest-neighbor search. Significance was determined by Fisher’s exact test. Asterisks denote statistical significance (***P < 0.001). (**E**) Spatial interaction heatmaps between pairs of marker-defined cell populations in *RNF43^wt^* samples (left) and *RNF43^659mut^* samples (right), restricted to the tumor region. Scores reflect the relative spatial colocalization or affinity between cell types, with greater values indicating closer interactions.

We further investigated the spatial dynamics of the *RNF43^659mut^* TME, and specifically tumor-NK cell interactions, by examining MSI-H CRCs (Cohort-3) with CyCIF, a highly multiplexed single-cell imaging method (*19*) (Fig. 5A). CyCIF imaging revealed increased expression of HLA-E and activation of the PI3K/AKT pathway (pAKT) in tumor cells (PanCK^+^) and stroma of *RNF43^659mut^* tumors (Fig. 5B, top and bottom, and fig. S9, A and B). When we quantified NK cell subsets in tumor regions, the overall frequency of CD56^+^CD3^−^ NK cells did not differ significantly between *RNF43^659mut^* and *RNF43^wt^* tumors, likely due to the small number of tumors in this cohort (Fig. 5C). However, we observed a trend toward reduced CD56^+^CD16^+^ functional NK cells in *RNF43^659mut^* tumors (Fig. 5C), a pattern consistent with impaired NK cytotoxic function. Notably, in *RNF43^659mut^* tumors, we observed infiltrating NKG2A^+^ NK cells in close proximity to HLA-E–expressing tumor cells (Fig. 5B, top and inset).

To quantify these findings, we measured local proximity by calculating the proportion of cells whose nearest neighbor resides within a fixed 20-μm radius using a two-nearest-neighbor search (see Materials and Methods). This proximity analysis demonstrated a significantly greater fraction of HLA-E^+^ tumor cells in *RNF43^659mut^* tumors having a NKG2A^+^ NK cell as their nearest neighbor within 20 μm than in wild-type tumors (Fig. 5D, top). In addition, when considering all nearest-neighbor distances between these cell types; NKG2A^+^NK cells and HLA-E^+^ cells had the strongest interaction score (0.69) in the *RNF43^659mut^* samples unlike in the *RNF43^wt^* ones (0.31), suggesting closer proximity of these two cell populations in the mutant tumors (Fig. 5E).

CD56^+^CD16^+^ functional NK cells in *RNF43^659mut^* tumors also showed closer spatial proximity to CD56^+^NKG2A^+^ NK cells (interaction score = 0.66) compared to *RNF43^wt^* (interaction score = 0.59) (Fig. 5E), suggesting that previously activated NK cells may become functionally exhausted when recruited into HLA-E–rich tumor niches. Likewise, pAKT^+^ tumor cells exhibited increased spatial coupling with HLA-E^+^ tumor cells (Fig. 5, B, bottom and insets, and D, bottom), with a higher spatial interaction score in *RNF43^659mut^* (0.47) compared to *RNF43^wt^* (0.18) (Fig. 5E), consistent with PI3K/AKT-driven up-regulation of HLA-E and the formation of coordinated immune-evasive epithelial states.

Collectively, these quantitative spatial analyses including marker density, tumor-immune proximity, and nearest-neighbor spatial interaction metrics demonstrate that *RNF43^659mut^* tumors create pAKT-high, HLA-E–enriched tumor regions that are preferentially infiltrated by NKG2A^+^ NK cells, reinforcing a PI3K/AKT-dependent HLA-E/NKG2A immune-checkpoint mechanism that suppresses NK cytotoxicity in CRC.

## DISCUSSION

This study elucidates the mechanisms by which the *RNF43 p.G659fs* mutation (*RNF43^659mut^*), which is enriched in MSI-H CRC (*13*), contributes to NK cell dysfunction and immune evasion in this disease. Through scRNA-seq and mIF analyses of patient-derived specimens, we observed that *RNF43^659mut^* tumors had increased NK cell infiltration, but in coculture experiments with isogenic CRC lines and PDOs, we found that these NK cells exhibited reduced maturation, functional impairment, and impaired cytotoxicity, underscoring the effect of this mutation on NK cell–mediated antitumor immunity. The tumor specimens, from previously characterized cohorts of patients with MSI-H CRC, represent advanced-stage disease and immune infiltration, providing a clinically relevant context in which *RNF43^659mut^*-mediated NK dysfunction occurs (*21*, *36*–*38*). Notably, we consistently observed elevated expression of HLA-E in association with *RNF43^659mut^* across multiple models. Mechanistically, we found that the activation of the PI3K/AKT/mTOR pathway promoted HLA-E up-regulation and contributed to NK cell immune resistance, as evidenced by restored NK cell cytotoxicity following pharmacological or genetic inhibition of this pathway. These findings were further supported by spatial analyses of MSI-H CRCs using CyCIF, which demonstrated elevated HLA-E expression, and increased PI3K/AKT pathway activity in the *RNF43^659mut^* CRC TME. Spatial proximity between NKG2A^+^ NK cells, HLA-E–expressing tumor cells, and pAKT-high tumor cells confirmed in situ that PI3K/AKT-driven HLA-E up-regulation mediates immune evasion via the HLA-E/NKG2A axis.

In addition to NK cells, we observed enrichment of ZBTB16^+^ (PLZF^+^) lymphocytes in *RNF43*^*659mut*^ CRC. PLZF is a key transcriptional regulator of innate-like lymphocyte programs, including invariant NKT cells and subsets of γδ T cells, which can share rapid effector and cytotoxic features with NK cells (*39*–*41*). Prior work in CRC has also linked PLZF expression with CD2/CD56^+^ immune populations, and NK/NKT-like cells have been shown to have spatial and prognostic relevance in the CRC microenvironment (*21*, *42*). Thus, enrichment of ZBTB16^+^ cells may reflect broader remodeling of innate-like immune compartments in *RNF43^659mut^* tumors. However, the functional contribution of this population remains to be determined in future studies.

Although we used an *RNF43^659mut^* overexpression system in HCT116 to complement the CRISPR-edited and patient-derived models, we acknowledge that forced expression may not fully recapitulate endogenous regulation of *RNF43^659mut^* or its allele-specific effects. The key phenotypes observed in the overexpression system—HLA-E up-regulation, PI3K/AKT pathway activation, and reduced NK cell cytotoxicity—were consistently reproduced in the CRISPR-edited HT29 and LS513 isogenic lines and in *RNF43^659mut^* PDOs, supporting their biological relevance. We therefore interpret the overexpression data as mechanistic reinforcement rather than primary evidence and highlight the endogenous and CRISPR-based models as the foundation for our central conclusions. In addition, we used interleukin-2 (IL-2)/IL-15 to stimulate healthy donor-derived NK cells. Although IL-2/IL-15–stimulated NK cells from healthy donors enabled controlled functional comparisons, they may not fully reflect the phenotype of tumor-infiltrating NK cells in vivo. Donor-to-donor variability was observed at the level of absolute cytotoxicity and during functional marker expression, but the *RNF43^659mut^*-dependent dysfunctional phenotype was consistent across all donors, supporting the robustness of our findings. To further contextualize these findings, our data support a mechanistic model in which *RNF43^659mut^* activates PI3K/AKT signaling, leading to selective up-regulation of HLA-E while concurrently dampening classical antigen presentation, NKG2D ligand expression, and interferon signaling, pathways that normally promote NK cell activation (*14*, *15*, *23*, *43*). Because HLA-E preferentially engages the inhibitory receptor NKG2A, its induction shifts NK cell signaling toward immune suppression and provides a direct explanation for why *RNF43^659mut^* tumor cells become resistant to NK-mediated cytotoxicity. Although NK cells can acquire inhibitory ligands through trogocytosis (*33*–*35*), our coculture assays demonstrated only modest and transient HLA-E transfer, suggesting that trogocytosis is not the primary driver of NK dysfunction; rather, *RNF43^659mut^*-mediated PI3K/AKT activation and tumor-intrinsic HLA-E up-regulation predominate. *HLA-E* knockout partially restores NK cell killing, demonstrating that HLA-E is not simply correlated with *RNF43^659mut^* status but serves as a functional mediator of immune evasion. As other nonclassical inhibitory ligands such as HLA-G were not detected in our models, these results indicate that HLA-E represents the dominant suppressive axis downstream of *RNF43^659mut^*-PI3K/AKT signaling. Thus, future preclinical and clinical studies combining PI3K/AKT/mTOR with HLA-E inhibition could be worth pursuing.

The PI3K/AKT/mTOR pathway plays a critical role in promoting immune suppression by modulating multiple cellular and molecular mechanisms (*15*, *43*–*46*), including up-regulation of immune checkpoint molecules like PD-L1 (*47*, *48*). In addition, PI3K/AKT signaling can impair antigen presentation by down-regulating classical major histocompatibility complex class I (MHC-I) molecules (HLA-A, HLA-B, and HLA-C), thereby limiting the recognition and clearance of tumor cells by cytotoxic lymphocytes (*49*). In contrast, nonclassical MHC-I molecules such as HLA-E, which interact with inhibitory NK cell receptors like NKG2A, may be differentially regulated and warrant exploration. Although PD-L1 induction and MHC-I down-regulation primarily affects T cell–mediated immunity, the HLA-E up-regulation we identify in this work could provide a parallel mechanism of immune escape by engaging with NKG2A on NK cells, thereby directly impairing innate cytotoxic responses (*23*, *50*, *51*). Although our data establish *RNF43^659mut^* as a driver of PI3K/AKT-dependent HLA-E up-regulation and NK cell dysfunction, these findings are also consistent with a broader model in which PI3K/AKT activation itself may promote HLA-E–mediated immune evasion across tumor contexts, with *RNF43^659mut^* representing a clinically relevant genetic mechanism in MSI-H CRC. Future studies will be required to determine the extent to which distinct PI3K pathway alterations similarly regulate HLA-E expression and NK cell resistance across tumor types. Together, these findings suggest that *RNF43^659mut^* tumors may leverage the PI3K/AKT/mTOR pathway to simultaneously suppress both innate and adaptive immune surveillance.

Inhibition of PI3K/AKT signaling using perifosine or siRNA targeting *AKT1* or *PIK3C2A* reduced HLA-E expression, restored NK cell activation, and enhanced tumor cell killing. Of note, the rescue of NK cell cytotoxicity observed with PI3K/AKT inhibition was more pronounced than that achieved by *HLA-E* knockout, supporting a model in which *RNF43^659mut^*-driven PI3K/AKT activation regulates HLA-E as a dominant inhibitory axis while also engaging additional immunosuppressive pathways. Dual blockade of PI3K/AKT and NKG2A signaling with perifosine and monalizumab, respectively, augmented NK cell cytotoxicity and tumor clearance in *RNF43^659mut^* models. Although most immunotherapy efforts in CRC have focused on T cell–based checkpoint blockade, particularly in MSI-H disease, NK cell–directed strategies are now emerging (*52*–*54*). These include cytokine-activated NK cells, chimeric antigen receptor–NK platforms, and agents that boost NK cytotoxicity; however, clinical efficacy has remained modest, potentially due to tumor-intrinsic suppressive mechanisms, possibly such as HLA-E/NKG2A-mediated inhibition. Our results suggest that rational combination approaches, particularly those pairing NK-activating therapies with targeted blockade of tumor escape pathways like PI3K/AKT/mTOR, may offer a promising therapeutic avenue. These data provide a strong mechanistic rationale for evaluating dual NK checkpoint and PI3K pathway blockade in *RNF43^659mut^* CRC and other immunosuppressive tumor phenotypes. In particular, given that the *RNF43^659mut^* mutation has been identified in other cancer types (*12*) and that other PI3K/AKT/mTOR mutations as well as PI3K pathway–driven immune resistance mechanisms are operant in CRC and other malignancies (*15*), our findings may inform broader combinatorial immunotherapy strategies across tumor types with shared molecular features.

In conclusion, our study identifies the PI3K/AKT/mTOR pathway as a viable therapeutic target in *RNF43^659mut^* MSI-H CRC. By reducing HLA-E expression and restoring NK cell functionality, pathway inhibition enhances innate immune responses and offers a strategy to potentiate existing immunotherapies. The identification of PI3K/AKT/mTOR pathway activation and HLA-E up-regulation as key drivers of NK cell dysfunction highlights the need for novel combinatorial approaches. Future studies should evaluate the integration of PI3K/AKT/mTOR inhibitors with immune checkpoint blockade and NK cell–targeted therapies to overcome immune resistance in this and other tumor types harboring mutations in the PI3K/AKT/mTOR pathway. Together, these findings provide a strong foundation for the development of precision immunotherapies targeting *RNF43^659mut^* and improving outcomes for patients with CRC.

## MATERIALS AND METHODS

### Tumor cell lines and culture

HT29 (RRID: CVCL_0320), HCT116 (RRID: CVCL_0291), and LS513 (RRID: CVCL_1386) were obtained from the American Type Culture Collection (ATCC; Manassas, VA, USA). All cell lines were authenticated by the ATCC and used at a low passage number from the original vial. Cell lines were cultured under standard conditions recommended by the manufacturer supplemented with 10% fetal bovine serum (Sigma-Aldrich, catalog no. F2442) and 1% penicillin/streptomycin (Thermo Fisher Scientific, catalog no. BW17603E) and kept at 37°C in a humidified atmosphere with 5% CO_2_. All cell lines were authenticated by validating short tandem repeat fingerprinting and routinely tested negative for Mycoplasma contamination. HT29 and LS513 CRISPR-Cas9 genome-edited isogenic cell lines were obtained from our previous study (*14*). The *RNF43^659mut_OE^* HCT116 line and CRISPR-Cas9 genome-edited organoid line (organoid 2) were generated as previously explained (*14*) with the same plasmid/virus constructs.

### Human specimens

ScRNA-seq data (Cohort-1; *n* = 33) and mIF (Cohort-2; *n* = 91) data from patients with MSI-H CRC were obtained from previous studies (*21*, *38*). Cohort-2 specimens were incident CRCs from two prospective cohort studies in the US: the Nurses’ Health Study (NHS) and the Health Professionals Follow-up Study (HPFS) (*21*). CyCIF was performed in MSI-H CRC specimens at baseline (Cohort-3; *n* = 14). Human colonic tissues and clinical data for Cohort-3 as well as for PDOs were obtained from patients with CRC at the Dana-Farber Cancer Institute following informed consent and IRB-approved protocols (DFCI #03-189 and #20-000). Patient clinical and pathology data for Cohort-3 and patient tissues used for PDO generation are listed in tables S1 and S2, respectively.

### Patient-derived colon organoid culture

To establish PDOs, colon tumor, metastatic tissues, and adjacent normal tissue specimens were obtained from surgical resections and one previously established mouse xenograft. Samples were minced and incubated with collagenase XI (5 mg/ml; MilliporeSigma, catalog no. C9407), DNase I (10 μg/ml; STEMCELL Technologies, catalog no. 07900), and 10 μM Y-27632 (MilliporeSigma, catalog no. Y0503). The dissociated cells were embedded in domes of growth factor reduced Matrigel (BME; R&D Systems, catalog no. BME001-05) in 24-well plates. After Matrigel solidification for 10 min at 37°C, cells were cultured in human intestinal tumor organoid medium composed of Advanced Dulbecco’s modified Eagle’s medium (DMEM)/F-12 (Gibco, catalog no. 12634010), 1X GlutaMAX, 10 mM Hepes, penicillin-streptomycin (100 U/ml; Life Technologies, catalog no. 15140122), 1X B-27 supplement (Thermo Fisher Scientific, catalog no. 17504044), Noggin (100 ng/ml; Thermo Fisher Scientific, catalog no. PHC1506), 1.25 mM *N*-acetylcysteine (MilliporeSigma, catalog no. A9165), 10 mM nicotinamide (MilliporeSigma, catalog no. N3376), 10 μM SB202190 (MilliporeSigma, catalog no. S7067), epidermal growth factor (50 ng/ml; Life Technologies, catalog no. PMG8041), 0.5 μM A 83-01 (Tocris, catalog no. 2939), and 1 μM prostaglandin E_2_ (Tocris, catalog no. 2296) or in human intestinal normal organoid medium [tumor organoid medium supplemented with 0.5 nM Wnt surrogate (Life Technologies, catalog no. PHG0401) and R-spondin-3 (50 ng/ml; VWR, catalog no. 0779-282)]. Media were changed every 2 to 3 days and confluent organoids were passaged by incubating in Gentle Cell Dissociation Reagent (STEMCELL Technologies, catalog no. 100-0485). PDOs were resuspended in a 1:1 mixture of Matrigel (BME) and basal media composed of DMEM/F-12 (Thermo Fisher Scientific, catalog no. 11320033), 10 mM Hepes (Life Technologies, catalog no. 15630080), bovine serum albumin (BSA) (1 mg/ml; Life Technologies, catalog no. BP1605), and penicillin-streptomycin (100 U/ml). During organoid establishment and passaging, Y-27632 (STEMCELL Technologies, catalog no. 72304) was included in the organoid growth media for 2 to 3 days until the media refreshment. Whenever necessary, PDOs were dissociated into single cells via enzymatic digestion in TrypLE Express (Thermo Fisher Scientific, catalog no. 12604013) for 10 min at 37°C. Established PDOs were profiled by whole-exome sequencing (WES), and the genomic characteristics of patient-derived specimens are listed in table S3.

### scRNA-seq and WES data analysis

WES on Cohort-1 specimens was performed using the GATK best practices as implemented in the Broad Cancer Genome Analysis Firecloud workflow (*55*, *56*). Briefly, after quality control (QC) and alignment with BWA, variant discovery (SNVs and indels) was performed using Mutect1 and Mutect2 (*57*) and Strelka (*58*). Variants were filtered for sequencing and sample preparation artifacts using OxoG (*59*) and a comprehensive panel of normal samples. Single-cell data from 33 MSI-H human colorectal tumors were obtained as previously described (*38*), and the mutational status of tumors including RNF43 mutational status, obtained through WES, is listed in table S4. Cell type counts were obtained from the Midway-Clustering level, with immune cells consisting of the following: B cells, dendritic cells, granulocytes, innate lymphoid cells, macrophages, mast cells, monocytes, NK cells, plasma cells, CD4^+^ T cells, CD8^+^ T cells, ZBTB16^+^ (PLZF^+^) T cells, and γδ T cells. Immune cell counts were then used to determine relative immune cell proportions for each tumor. R version 4.3.0 was used for downstream analysis. Differences between MSI-H *RNF43^wt^* and *RNF43^659mut^* tumors were assessed using two-sided Mann-Whitney *U* tests, with a significance threshold of *P* value < 0.05. Differential expression analysis was performed between *RNF43^wt^* and *RNF43^659mut^* using the Seurat R package version 5.1.0, with significance thresholds of *P* (adjusted) value < 0.05 and |log_2_ fold change| > 0.75. Any statistically significant genes present in our manually curated lists of NK and epithelial cell markers were included in the heatmap. For heatmap representation, counts were aggregated to create pseudobulk expression profiles at a tumor level. Immune marker genes for immune profiling have been explained in (*38*).

### mIF data analysis

mIF for Cohort-2 specimens has been previously published (*21*). Briefly, to examine NK cells and other lymphocytic populations in the TME, custom mIF panels were used with the tyramide signal amplification method. Slides were scanned using the Vectra Polaris Automated Quantitative Pathology Imaging System (Akoya Biosciences), equipped with seven imaging filters and a 20x objective. NK cells were defined as PTPRC^+^NCAM1^+^CD3^−^ and T cells as PTPRC^+^NCAM1^−^CD3^+^. PTPRC (CD45) was used to identify pan-leukocytes, NCAM1 (CD56) to label NK cells, KRT (keratin) to mark epithelial cells, and 4′,6-diamidino-2-phenylindole (DAPI) as a nuclear counterstain. MHC-I antigen presentation molecules [B2M or HLA-A/HLA-B/HLA-C (*21*)] were also analyzed. MSI, TMB, and *RNF43* mutational statuses for these specimens were obtained previously (*37*). MSI-H samples were split into two groups (*RNF43^wt^* and *RNF43^659mut^*), and NK cell and tumor cell densities (cells/mm^2^) were compared in three localizations (tumor epithelium, stroma, and overall). Two-sided Mann-Whitney *U* tests were used for statistical testing using R version 3.6.0, with a significant threshold of *P* value < 0.05. Following the same methodology, HLA-ABC and B2M expression along with TMB were compared between MSI-H *RNF43^wt^* and *RNF43^659mut^* samples.

### Cyclic immunofluorescence

CyCIF staining of the tissues was performed following previously described protocols (*19*). In brief, 5-μm FFPE CRC sections were dewaxed on a BOND RX autostainer (Leica Biosystems, Buffalo Grove, IL, USA), and antigen retrieval was performed with BOND Epitope Retrieval Solution 1 buffer (Leica Biosystems, catalog no. AR9961) for 20 min. After dewaxing and antigen retrieval, the slides were immediately bleached with 4.5% H_2_O_2_ with LED (light-emitting diode) light to reduce autofluorescent background. The antibodies were incubated overnight at 4°C. After washing, the slides were imaged with a CyteFinder scanning fluorescence microscope (RareCyte Inc., Seattle, WA) using 20x objectives. The multiple-cycle images were achieved via repetitive antibody incubation, imaging, and fluorophore inactivation (bleaching). For CyCIF image processing, after the raw images were acquired, image stitching and registration were performed using the ASHLAR module (*60*) in the Docker-based NextFlow pipeline MCMICRO (*61*) to assemble OME.TIFF images. MCMICRO was subsequently used to segment images and quantify cell fluorescence intensities (*62*). For QC of the single-cell data of the multiplexed images, CyLinter was used (*63*) to remove image-processing and segmentation artifacts. To generate binary gates for cell type identification, assign cell types based on lineage-specific marker expression, and downstream analysis, customized scripts were used in Python, R, and MATLAB. All samples were gated independently using two-component Gaussian mixture modeling and validated by visual inspection. All antibodies used for CyCIF are listed in table S5.

### Spatial interaction and proximity analysis

Spatial interaction between pairs of marker-defined cell populations was quantified using a symmetric nearest-neighbor ratio metric comparing observed and randomized cell-cell distances. For each slide and each marker pair (A, B), all A^+^ and B^+^ cells were identified on the basis of marker positivity within the annotated tissue region. To measure spatial proximity, we computed pairwise nearest-neighbor distances using *K*-nearest-neighbor search. The Euclidean distance between A^+^ to the nearest B^+^ neighbor was computed; similarly, for B^+^ cells, nearest A^+^ nearest-neighbor distance was computed. To obtain an expected null distance distribution, cell positions were randomized by sampling an equal number of cells from the slide and recomputing nearest-neighbor distances. A symmetric interaction score was then computed as

$$\text{Interaction}\left(A,B\right)=\frac{\text{mean}\left(d_{A\to B}^{\text{rand}}\right)\cdot \text{mean}\left(d_{B\to A}^{\text{rand}}\right)}{\text{mean}\left(d_{A\to B}^{\text{obs}}\right)\cdot \text{mean}\left(d_{B\to A}^{\text{obs}}\right)}$$

Spatial interaction scores were interpreted as a continuous measure of relative spatial affinity, with larger values indicating closer-than-average proximity between cell populations. Next, local proximity between cell populations was quantified by measuring the fraction of HLA-E^+^ cells whose nearest marker (i.e., pAKT^+^ or CD56^+^ NKG2A^+^) neighbor lies within a fixed 20-μm radius using a two-nearest-neighbor search (the second neighbor was used to avoid self-matches). The Euclidean distance to this neighbor was recorded, and the proportion of HLA-E^+^ cells with a positive neighbor within 20 μm was computed. To assess enrichment over random expectation, a null model was constructed by sampling an equal number of random cells from the same slide and recomputing nearest-neighbor distances, yielding a randomized proximity fraction. Observed and randomized fractions were computed for each marker pair and each slide. Differences in proximity fraction between groups were evaluated using Fisher’s exact test.

### NK cell isolation from healthy donor blood

Healthy donor blood was obtained from apheresis leukoreduction collars under the blood collection protocol approved by the Institutional Review Board of Brigham and Women’s Hospital. Peripheral blood mononuclear cells (PBMCs) were isolated using Ficoll-Paque density gradient separation, which was then cryopreserved until later or used immediately for NK cell isolation. NK cells were purified using the EasySep Human NK Cell Isolation Kit (STEMCELL Technologies, catalog no. 17955), and purity was checked by flow cytometry (>95% CD56^+^CD3^−^). Then, NK cells were mildly stimulated for 22 to 24 hours at 37°C in RPMI 1640 medium (GIBCO, catalog no. 11875093) supplemented with human IL-15 Recombinant Protein (PeproTech, catalog no. 200-15-10UG; 10 ng/ml) and human IL-2 Recombinant Protein (PeproTech, catalog no. 200-02-100UG; 100 IU/ml). We did not observe a difference in the activity of NK cells, whether freshly isolated or isolated from cryopreserved PBMCs.

### Tumor-NK cell coculture

To measure NK cell functional states in cocultures with CRC cell lines, 2 × 10^4^ tumor cells were seeded in 96-well assays and cultured for 24 hours in the respective tumor line culture media. After 24 hours, the media were refreshed, and healthy donor NK cells were added while maintaining a 1:5 target-to-effector (T:E) cell ratio. The cells were then cocultured for 24 hours before being analyzed by flow cytometry. For PDOs, confluent organoids were harvested and dissociated into single cells using TrypLE Express. Ninety-six-well plates were first coated with BME (1:20 in cold DMEM) by adding 100 μl per well and incubating for 30 to 45 min at 37°C. The DMEM was then carefully removed without disturbing the BME layer and replaced with warm organoid growth media supplemented with Y-27632, and 20,000 suspended single-cell PDOs were seeded per well. PDOs were incubated for 24 to 48 hours, after which the media were refreshed before adding NK cells while maintaining a 1:5 T:E cell ratio. PDOs from a single well were dissociated into single cells and recounted to confirm the target cell number using hemocytometer. After 24 hours of coculture at 37°C, cells were collected from wells and dissociated into single cells.

To measure NK cell cytotoxicity against cell lines, 1 × 10^5^ tumor cells were seeded in 48-well plates and cultured for 24 hours before being cocultured with NK cells. For PDOs, the 48-well plates were first coated with BME (1:20 in cold DMEM, 200 μl), after which single-cell suspensions of PDOs in growth media supplemented with Y-27632 were layered on the BME and allowed to grow for 24 to 48 hours. The organoid growth media were then refreshed, and PDOs from a single well were recounted before adding NK cells. After 24 hours of coculture, all cells in the wells were collected, dissociated into single cells using TrypLE Express, and analyzed by flow cytometry. For all experiments, a T:E ratio of 1:5 was maintained. The T:E ratio of 1:5 was determined through optimization assays performed with different ratios (1:1, 1:5, and 1:10).

### Multicolor flow cytometry–based functional assays, trogocytosis, and cytotoxicity assays

To determine NK cell functional states in tumor-NK cocultures of CRC cell lines and PDOs, single-cell suspensions were first stained with surface markers with CD56, CD3, CD16, NKG2D, NKG2A, KIR3DL1, CD158a, TIM3 antibodies (BioLegend), and Fixable Viability Dye eFluor 780 (eBioscience, catalog no. 65-0865-14) to exclude dead cells before to analyze intracellular markers. To estimate intracellular functional markers (IFN-γ and TNFα), at 20 hours of tumor-NK coculture, Golgi-Plug (1:1000, BD Bioscience, catalog no. 555029) and Golgi-Stop (1:1500, BD Bioscience, catalog no. 554724) were added to the coculture and incubated for another 4 hours. After staining for surface markers with the manufacturers’ suggested dilutions of antibodies, intracellular labeling was done by fixing and permeabilizing the cells with the BD Cytofix/Cytoperm Fixation/Permeabilization Solution Kit (BD Biosciences, catalog no. 554714) following the manufacturer’s protocol and with IFN-γ and TNFα antibodies (R&D Systems).

To analyze NK cell cytotoxicity, single-cell suspensions of tumor-NK cell cocultures of CRC cell lines and PDOs were incubated with the manufacturer’s recommended dilution of fluorescently labeled antibodies for CD45 and EpCAM (CD326; BioLegend) before staining with the FITC Annexin V Apoptosis Detection Kit with Propidium Iodide (BioLegend). This combination allows for the separation of NK cells from tumor cells. Propidium iodide (PI) and annexin V–fluorescein isothiocyanate (FITC) staining were performed according to the manufacturer’s protocol. To assess trogocytosis, NK cells were cocultured with HT29 *RNF43* isogenic tumor lines or organoid 3 for 0, 1, 4, and 24 hours. At each time point, cells were collected and analyzed by flow cytometry to measure HLA-E and HLA-G expression on both NK cells and tumor cells. NK cells were identified using CD45^+^CD56^+^CD16^+^ gating during flow cytometry analysis, and tumor cells were gated using EpCAM^+^ expression. All fluorescently labeled cells were acquired on an LSR Fortessa (BD Biosciences) and analyzed using the FlowJo software (version 10, BD Biosciences). Before staining with the relevant antibody panels, NK cells were incubated with an Fc receptor blocking reagent for 10 min to prevent nonspecific antibody binding (BD Biosciences, catalog no. 564219). All antibodies and isotype controls used for flow cytometry are listed in table S6.

### Coculture cell sorting and RNA-seq

NK cells and HT29 isogenic tumor cells were sorted with gating on CD45^+^CD56^+^ and CD45^−^EpCam^+^ cells from NK-HT29 cocultures at 24 hours of coculture using Symphony S6 (BD Biosciences). Total RNA was extracted from sorted samples using the RNeasy Plus Mini Kit (Qiagen, catalog no. 74104). RNA integrity was measured using Bioanalyzer (Agilent). RNA-seq of the sorted NK cells and HT29 tumor cells was performed by MedGenome Corporation Inc. Service using the Illumina NovaSeq with an average aligned read count of 37 million for NK cells and 36 million for HT29 cells. Paired-end reads were aligned to the human genome reference build GRCh38/hg38 using STAR version 2.7.3a for NK cells and 2.7.11b for HT29 tumor cells. Differential expression analysis was performed using R version 3.6.0 and the DESeq2 R package version 1.42.1, correcting for donor variation. The significance threshold was set at *P* adjusted value < 0.05. Any statistically significant genes present in our manually curated lists of NK and HT29 tumor cell markers were included in the heatmaps. For heatmap representation, variation caused by different donors was removed using the limma R package version 3.58.1.

### Construction of GFP- or mCherry-expressing tumor lines

Lentivirus was produced by transfecting human embryonic kidney (HEK) 293T cells with psPAX2 (RRID: Addgene_12260) and VSV-G (RRID: Addgene_8454) packaging plasmids, along with LV-GFP (RRID: Addgene_25999) or pLenti6-H2B-mCherry (RRID: Addgene_89766). Stable transfection was performed in the indicated cell lines via lentiviral transduction, followed by a 48-hour incubation period. For organoid lines, lentiviral transduction was conducted in single-cell suspensions without BME. After incubation with the virus, single cells were collected by centrifugation and subsequently embedded in BME domes as described earlier. These single cells were then cultured into PDOs over a period of 2 to 3 weeks. Once confluent, GFP- or mCherry-expressing cells were sorted by FACS. The sorted cells were then collected by centrifugation and reseeded into BME domes, where they were allowed to grow until reaching confluency (another 2 to 3 weeks).

### Wide-field fluorescence live imaging

Live-cell wide-field fluorescence imaging was performed using the THUNDER Imager 3D Cell Culture system (Leica Microsystems). PDOs expressing mCherry, or isogenic HT29 cell lines expressing mCherry or GFP, and cocultured with NK cells were imaged after 24 hours of incubation at 10× magnification. Image analysis was carried out using the ImageJ software (NIH).

### siRNA-mediated PI3K/AKT pathway inhibition

Human siRNA oligo duplexes for AKT1 (Locus ID: 207; catalog no. SR319307) and PI3K Class 2A (PIK3C2A; Locus ID: 5286; catalog no. SR321311) were purchased from OriGene Technologies Inc. A pooled cocktail of three unique 27-mer siRNA duplexes for each gene was used, with a final siRNA concentration of 3 pmol per well in a 48-well plate containing 1 × 10^5^ cells. siRNA transfections were performed using Lipofectamine RNAiMAX Reagent (Life Technologies, catalog no. 13778100) according to the manufacturer’s guidelines. For HT29 isogenic cell lines, 1 × 10^5^ cells were seeded in 48-well plates and cultured for 24 hours. The media were refreshed before adding target or scramble siRNA, followed by incubation for 48 hours. After incubation, cells were washed three times with fresh media before adding NK cells (T:E ratio of 1:5). The cocultures were maintained for 24 hours before flow cytometry analysis. For PDOs, organoids were dissociated into single cells, and 1 × 10^5^ cells were incubated with target or scramble siRNA complexes for 48 hours. Cells were then collected, washed three times in organoid growth media, and layered onto BME (1:20 in cold DMEM)-coated wells in a 48-well plate. PDOs were allowed to grow for 24 hours, after which cells were recounted from a single well before adding NK cells (T:E ratio of 1:5). The cocultures were then incubated for another 24 hours and analyzed by flow cytometry.

### PI3K/AKT inhibitor treatments

To determine a sublethal dose of PI3K/AKT inhibitors, HT29 isogenic cell lines and PDOs were treated with perifosine (MedChemExpress, catalog no. HY-50909) (*31*, *64*) using a twofold dilution series from 0.5 to 20 μM. Cells were treated for 72 hours before assessing viability. For two-dimensional (2D) cultures, 2000 cells per well were seeded in 96-well plates and allowed to grow 24 hours before drug treatment. For organoid drug treatment experiments, PDOs were dissociated into single cells and resuspended in organoid growth media containing 10% BME (2000 cells per well). Cell viability was determined using the CellTiter-Glo 2.0 Cell Viability Assay (Promega, catalog no. G9242) for 2D cultures and the CellTiter-Glo 3D Cell Viability Assay (Promega, catalog no. G9681) for 3D cultures, following the manufacturer’s instructions. Luminescence was measured using a SpectraMax M5 microplate luminometer (Molecular Devices). Each assay was conducted in three independent experiments, each comprising at least three technical replicates. Luminescence output was normalized to untreated controls. Following determination of sublethal doses, perifosine at 1, 2, and 4 μM was tested in NK cell coculture for 4, 24, and 48 hours. For both CRC cell lines and organoids, 1 × 10^5^ cells per well were seeded in 48-well plates for 24 hours and single cells were spread onto BME-coated wells (1:20 dilution in cold DMEM) and cultured for 24 to 48 hours, respectively, before drug treatment. Following drug treatment, the compounds were removed by washing three times with the relevant media before adding NK cells. After perifosine treatment (2 and 4 μM) for 4 hours, NK cells were cocultured with the treated cells for an additional 24 hours. In some experiments, tumor cells were treated with perifosine in combination with monalizumab (20 μM; humanized anti-NKG2A blocking mAb; MedChemExpress, catalog no. HY-P99032) or with monalizumab alone, which was added immediately upon NK cell coculture.

### CRISPR-Cas9–mediated *HLA-E* knockout

For CRISPR-Cas9–mediated *HLA-E* knockout, experiments were performed using the Lipofectamine CRISPRMAX Reagent (Life Technologies, catalog no. CMAX00008) and *HLA-E* multi-guide gene knockout kit (EditCo Bio Inc., Redwood City, CA, USA) according to the manufacturers’ guidelines.

For HT29 isogenic cell lines, 1 × 10^5^ cells were seeded into 24-well plates and cultured for 24 hours before transfection. Fresh media were added, followed by transfection with *HLA-E* single guide RNA (sgRNA) or a universal control sgRNA (sgCTRL), and cells were incubated for 48 hours. After incubation, cells were washed and supplied with fresh media and then expanded sequentially into 6-well plates and 25-cm^2^ culture flasks.

For PDO experiments, organoid 3 was dissociated into single cells, and 1 × 10^5^ cells were incubated with *HLA-E* sgRNA or sgCTRL complexes for 48 hours. Cells were then collected, washed three times with organoid growth media, and replated into BME domes. PDOs were allowed to grow to confluence over 3 to 6 weeks. Gene-editing efficiency was confirmed by immunoblotting.

### Western blot analysis

Total proteins were extracted from 2D or 3D tumor models by lysis in Pierce RIPA lysis buffer (Thermo Fisher Scientific, catalog no. 89900) supplemented with protease and phosphatase inhibitor (Thermo Fisher Scientific, catalog no. PI78441). The Pierce BCA Protein Assay Kit (Thermo Fisher Scientific, catalog no. 23225) was used to quantify the protein concentration. Equal amounts of protein samples (20 μg) were loaded and separated on 4 to 20% polyacrylamide gel (Mini-PROTEAN TGX, Bio-Rad, catalog no. 4561094) at 200 V for 15 to 20 min. Subsequently, the gel was blotted on a 0.45-μm PVDF (polyvinylidene difluoride) transfer membrane (Millipore, catalog no. IPVH00010) for 1 hour at 100 V. Thereafter, the membranes were blocked with 5% BSA (Sigma-Aldrich) for 1 hour. After blocking, the membranes were incubated with primary antibody dilutions (table S7) in 5% BSA in 1X phosphate-buffered saline (PBS) (Corning, catalog no. 21-040-CM) for 12 to 18 hours at 4°C. Membranes were washed with 1X PBS and incubated for 2 hours with secondary antibodies (table S7) labeled with horseradish peroxidase in 1X PBS and 5% BSA. The Precision Plus Protein Dual Color molecular weight marker (Bio-Rad, catalog no. 1610374) was used to estimate the protein size after developing with the Pierce ECL Western Blotting Substrate (Thermo Fisher Scientific, catalog no. 32209). Protein marker expressions of each blot were quantitated using the Bio-Rad Image Lab software.

### Statistical analysis

All results are reported as mean ± SD unless otherwise specified. Statistical tests were carried out using GraphPad Prism version 10 (GraphPad Software Inc., La Jolla, CA, USA). Specific statistical tests used for each experiment are indicated in the corresponding figure legends. Depending on data distribution and experimental design, either parametric tests [one-way analysis of variance (ANOVA) followed by Tukey’s multiple-comparisons test] or nonparametric tests (Kruskal-Wallis followed by Dunn’s multiple-comparisons test) were used. Statistical significance was defined as *P* < 0.05.
