# Supplementary material for: RNF43 p.G659fs leads to natural killer cell dysfunction in MSI-high colorectal cancer through PI3K/AKT/mTOR signaling and HLA-E up-regulation
Source: Sci Adv. 2026 Sep 23;12(39):eadz6907. doi: 10.1126/sciadv.adz6907 (PMC13600164; doi:10.1126/sciadv.adz6907)
Supplement: Supplementary file 1 [file sciadv.adz6907_sm.pdf]

Science Advances
AAAS

Supplementary Materials for

RNF43 p.G659fs leads to natural killer cell dysfunction in MSI-high colorectal cancer through PI3K/AKT/mTOR signaling and HLA-E up-regulation

Pushpamali De Silva *et al.*

Corresponding author: Marios Giannakis, marios_giannakis@dfci.harvard.edu

Sci. Adv. **12**, eadz6907 (2026)
DOI: 10.1126/sciadv.adz6907

This PDF file includes:

Figs. S1 to S9
Tables S1 to S7

Figure S1

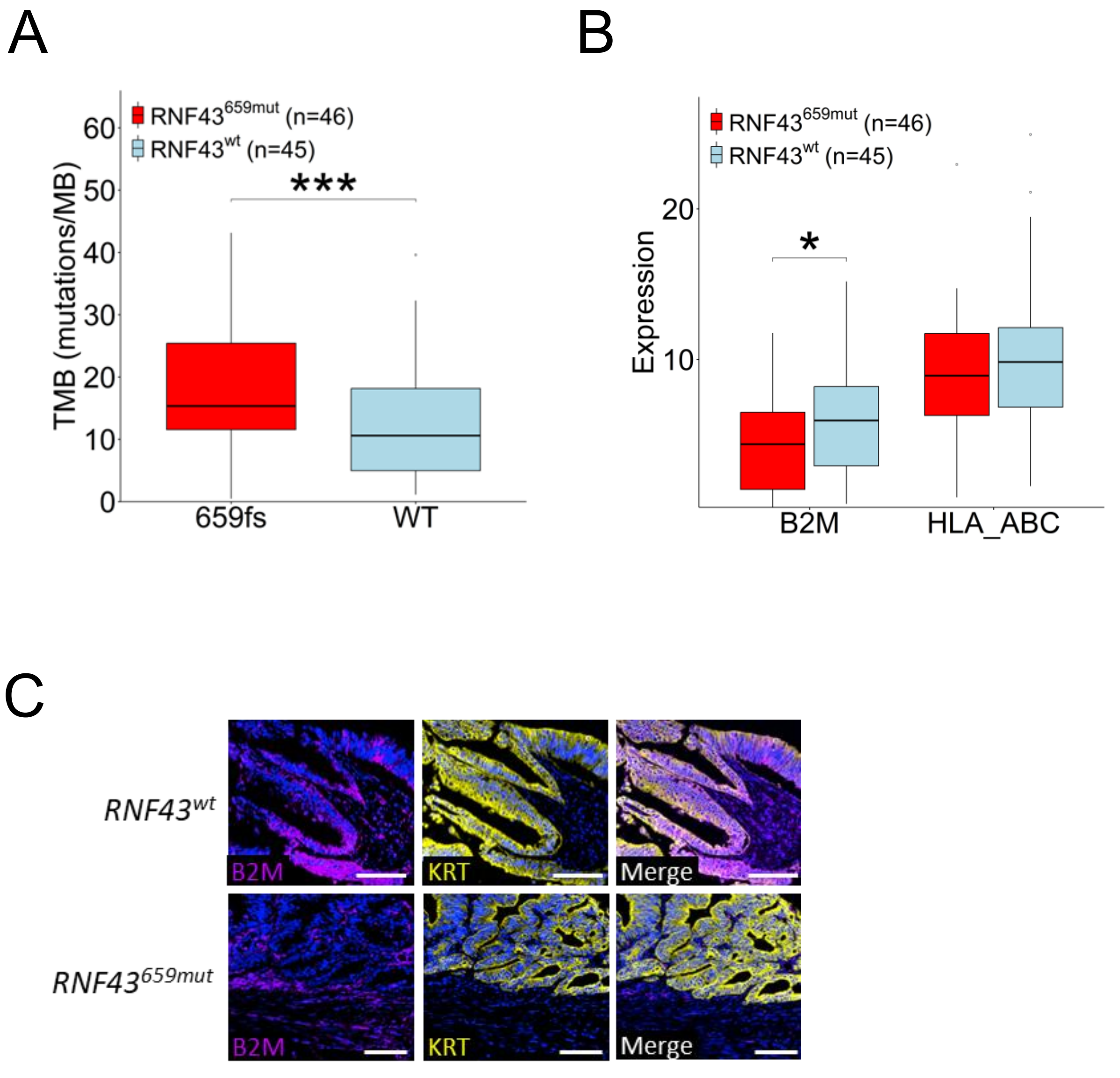

Figure S1. Genomic Landscape of *RNF43*^{659mut}. (A) Comparison of the tumor mutational burden (TMB) between *RNF43*^{659mut} and RNF43^{wt} MSI-H tumors from Cohort-2. TMB was derived from whole exome sequencing (*39*) performed in *RNF43*^{wt} tumors (n= 45) and *RNF43*^{659mut} tumors (n = 46). **(B)** Expression of *HLA-A*, *HLA-B* and *HLA-C* (HLA_ABC) and B2M in *RNF43*^{659mut} (n=46) versus *RNF43*^{wt} (n=45) tumors. HLA genes and B2M expression were analyzed using tissue microarray data obtained from (*21*). (C) Representative multiplex immunofluorescence images showing beta-2 microglobulin (B2M) expression in *RNF43*^{wt} and *RNF43*^{659mut} tumors. KRT – Keratin. For figures **(A)** and **(B)**, significance was determined by Mann-Whitney U test. Asterisks denote statistical significance (*P<0.05; ***P<0.001).

Figure S2

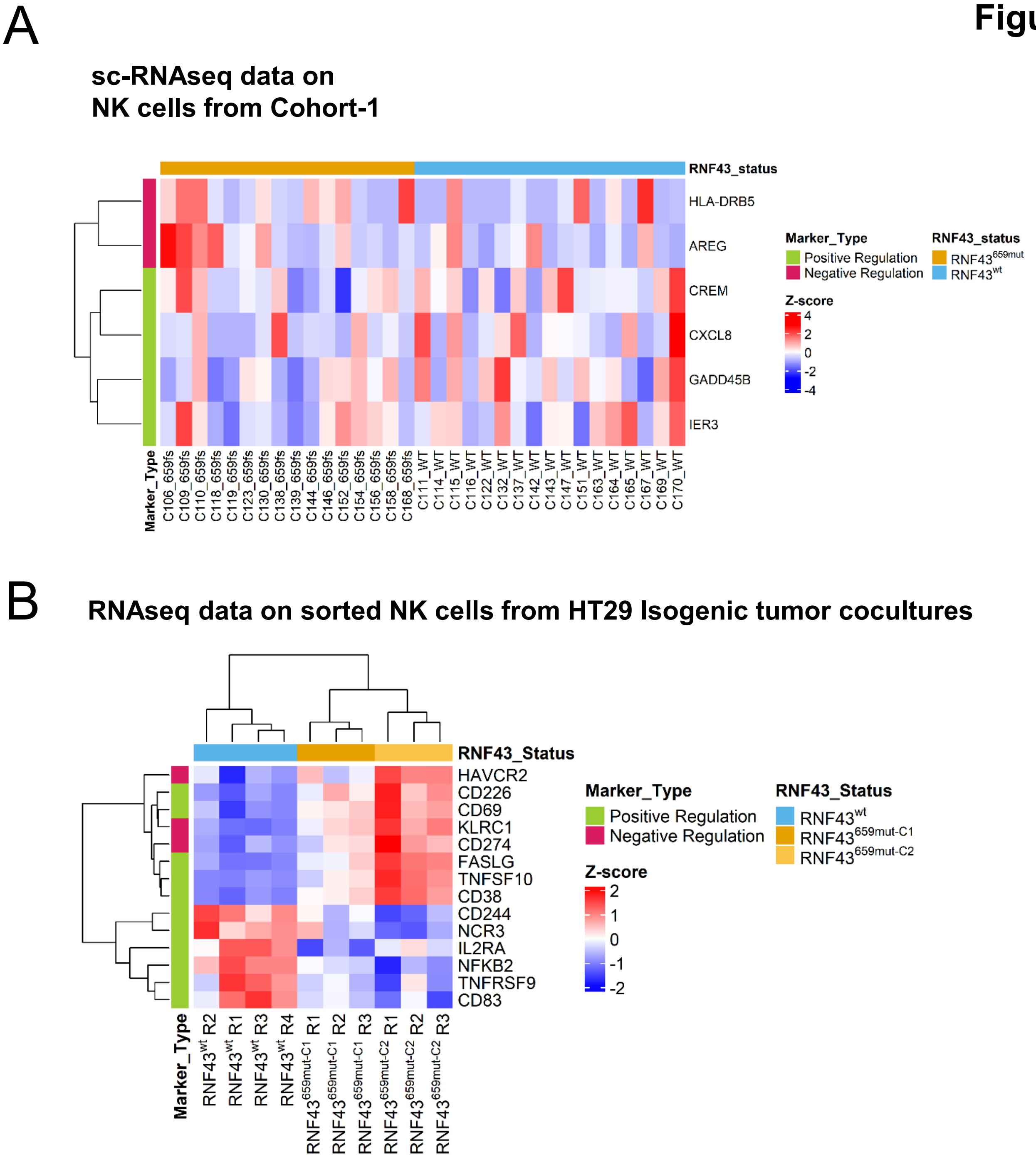

Figure S2. ***RNF43*^{659mut} induces transcriptional reprogramming of NK cells toward an exhausted phenotype. (A)** Single-cell RNA sequencing (sc-RNAseq) analysis of NK cells from Cohort-1 MSI-H CRCs. **(B)** Bulk RNA sequencing of FACS-sorted NK cells cocultured with HT29 isogenic tumor lines. The *RNF43*^{wt} line represents the wild-type HT29 cells, while RNF43$^{659mut\text{-}C1}$ and RNF43$^{659mut\text{-}C2}$ are two distinct mutant isogenic clones. R in each category denotes a biological replicate. For figures **(A)** and **(B)**, green marker type bar indicates activation markers that enhance NK cell activation and cytotoxic potential, while pink marker type bar represents inhibitory receptors that contribute to suppression of NK cell activation and effector functions in the tumor microenvironment. For figures **(A)** and **(B)** statistical significance was determined using the Seurat (cutoffs: padj < 0.05 and |log$_2$foldchange| > 0.75) and Deseq2 (cutoffs: padj < 0.05) R packages respectively.

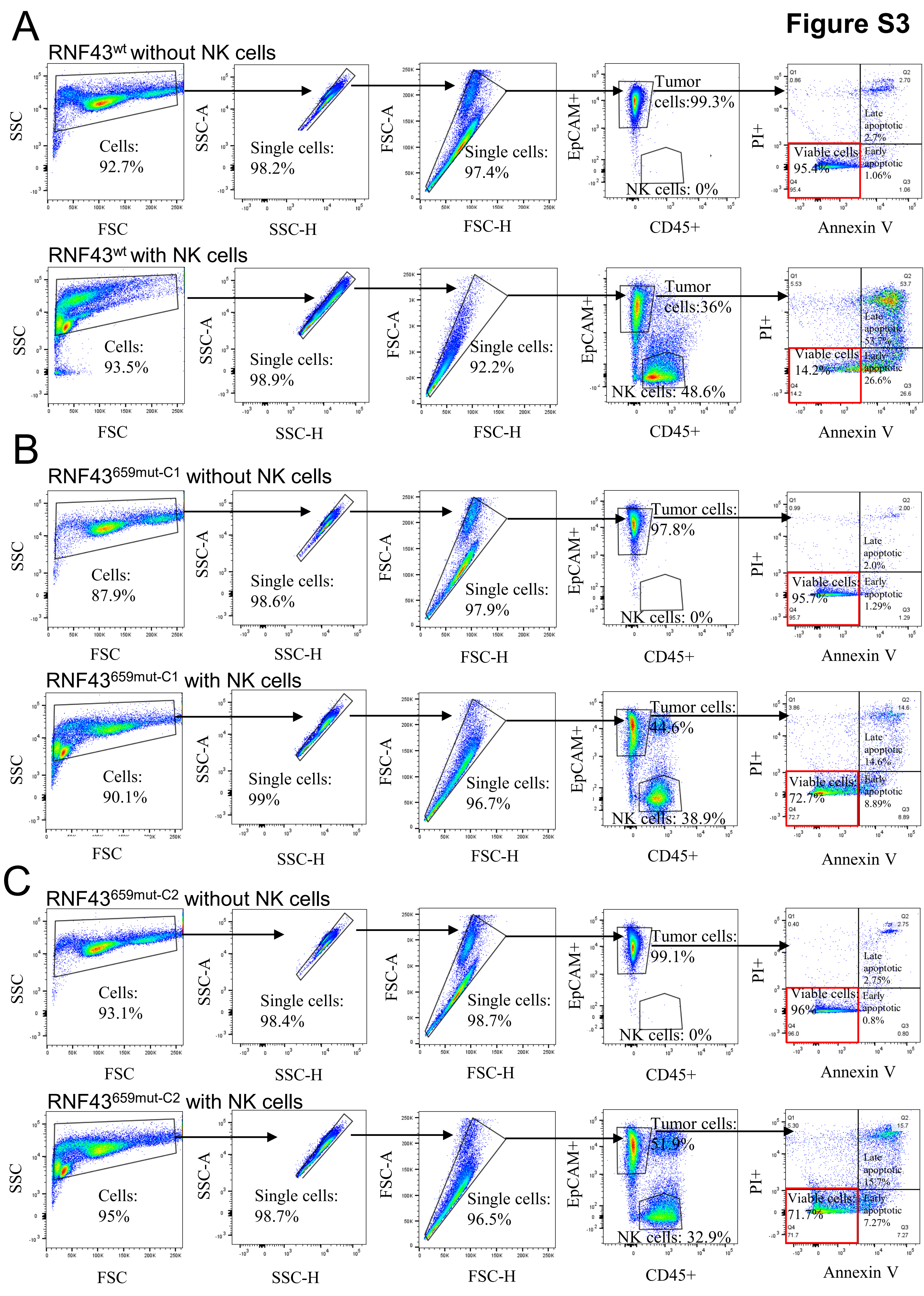

Figure S3
A
RNF43wt without NK cells
Cells: 92.7%
Single cells: 98.2%
Single cells: 97.4%
Tumor cells:99.3%
NK cells: 0%
Viable cells 95.4%
Early apoptotic 1.06%
Late apoptotic 2.7%
RNF43wt with NK cells
Cells: 93.5%
Single cells: 98.9%
Single cells: 92.2%
Tumor cells:36%
NK cells: 48.6%
Viable cells 14.2%
Early apoptotic 26.6%
Late apoptotic 53.7%
B
RNF43659mut-C1 without NK cells
Cells: 87.9%
Single cells: 98.6%
Single cells: 97.9%
Tumor cells: 97.8%
NK cells: 0%
Viable cells: 95.7%
Early apoptotic 1.29%
Late apoptotic 2.0%
RNF43659mut-C1 with NK cells
Cells: 90.1%
Single cells: 99%
Single cells: 96.7%
Tumor cells: 44.6%
NK cells: 38.9%
Viable cells: 72.7%
Early apoptotic 8.89%
Late apoptotic 14.6%
C
RNF43659mut-C2 without NK cells
Cells: 93.1%
Single cells: 98.4%
Single cells: 98.7%
Tumor cells: 99.1%
NK cells: 0%
Viable cells: 96%
Early apoptotic 0.8%
Late apoptotic 2.75%
RNF43659mut-C2 with NK cells
Cells: 95%
Single cells: 98.7%
Single cells: 96.5%
Tumor cells: 51.9%
NK cells: 32.9%
Viable cells: 71.7%
Early apoptotic 7.27%
Late apoptotic 15.7%
SSC
FSC
SSC-A
SSC-H
FSC-A
FSC-H
EpCAM+
CD45+
PI+
Annexin V

Figure S3. Flow cytometry gating strategy to assess NK cell-mediated killing in CRC models. Representative flow cytometry plots to analyze NK cell-mediated killing in HT29 **(A)** *RNF43^wt^*, **(B)** $RNF43^{659mut\text{-}C1}$ and **(C)** $RNF43^{659mut\text{-}C2}$ using NK cells isolated from the same donor. The same gating strategy was used to analyze NK cells killing in HCT116, LS513 tumor lines and CRC PDOs. Propidium iodide (PI) staining was used to identify dead cells, while Annexin V staining was used to estimate the percentage of apoptotic cells

Figure S4

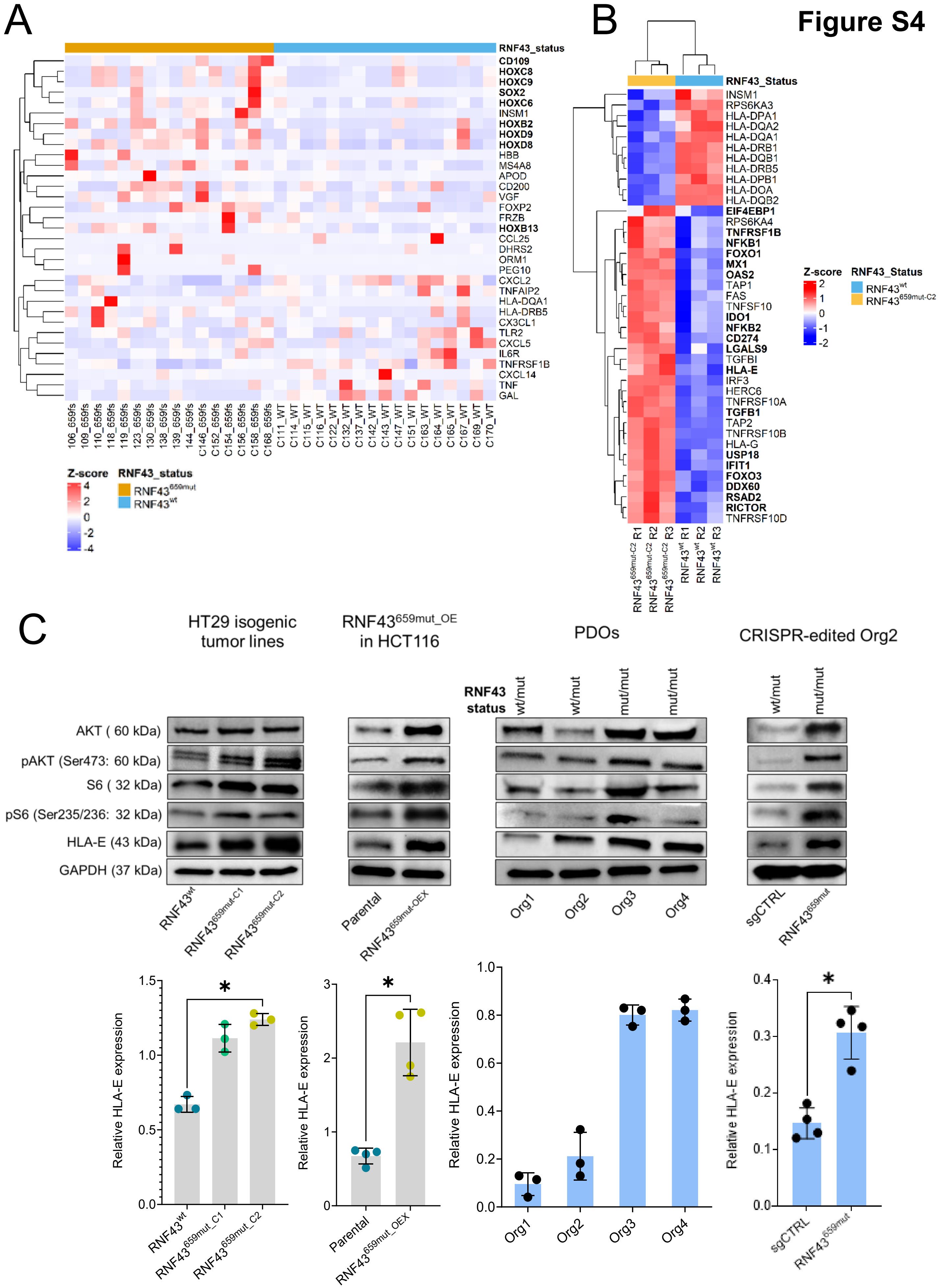

A
RNF43_status
CD109
HOXC8
HOXC9
SOX2
HOXC6
INSM1
HOXB2
HOXD9
HOXD8
HBB
MS4A8
APOD
CD200
VGF
FOXP2
FRZB
HOXB13
CCL25
DHRS2
ORM1
PEG10
CXCL2
TNFAIP2
HLA-DQA1
HLA-DRB5
CX3CL1
TLR2
CXCL5
IL6R
TNFRSF1B
CXCL14
TNF
GAL
106_659fs
109_659fs
110_659fs
118_659fs
119_659fs
123_659fs
130_659fs
138_659fs
139_659fs
144_659fs
C146_659fs
C152_659fs
C154_659fs
C156_659fs
C158_659fs
C168_659fs
C111_WT
C114_WT
C115_WT
C116_WT
C122_WT
C132_WT
C137_WT
C142_WT
C143_WT
C147_WT
C151_WT
C163_WT
C164_WT
C165_WT
C167_WT
C169_WT
C170_WT
Z-score
4
2
0
-2
-4
RNF43_status
RNF43659mut
RNF43wt
B
RNF43_Status
INSM1
RPS6KA3
HLA-DPA1
HLA-DQA2
HLA-DQA1
HLA-DRB1
HLA-DQB1
HLA-DRB5
HLA-DPB1
HLA-DOA
HLA-DQB2
EIF4EBP1
RPS6KA4
TNFRSF1B
NFKB1
FOXO1
MX1
OAS2
TAP1
FAS
TNFSF10
IDO1
NFKB2
CD274
LGALS9
TGFBI
HLA-E
IRF3
HERC6
TNFRSF10A
TGFB1
TAP2
TNFRSF10B
HLA-G
USP18
IFIT1
FOXO3
DDX60
RSAD2
RICTOR
TNFRSF10D
Z-score
2
1
0
-1
-2
RNF43_Status
RNF43wt
RNF43659mut-C2
RNF43659mut-C2 R1
RNF43659mut-C2 R2
RNF43659mut-C2 R3
RNF43wt R1
RNF43wt R2
RNF43wt R3
C
HT29 isogenic tumor lines
RNF43659mut_OE in HCT116
PDOs
CRISPR-edited Org2
RNF43 status
wt/mut
wt/mut
mut/mut
mut/mut
wt/mut
mut/mut
AKT (60 kDa)
pAKT (Ser473: 60 kDa)
S6 (32 kDa)
pS6 (Ser235/236: 32 kDa)
HLA-E (43 kDa)
GAPDH (37 kDa)
RNF43wt
RNF43659mut-C1
RNF43659mut-C2
Parental
RNF43659mut-OEX
Org1
Org2
Org3
Org4
sgCTRL
RNF43659mut
Relative HLA-E expression
1.5
1.0
0.5
0.0
*
RNF43wt
RNF43659mut_C1
RNF43659mut_C2
3
2
1
0
*
Parental
RNF43659mut_OEX
1.0
0.8
0.6
0.4
0.2
0.0
Org1
Org2
Org3
Org4
0.4
0.3
0.2
0.1
0.0
*
sgCTRL
RNF43659mut

Figure S4. Activation of the PI3K/AKT/mTOR pathway and HLA-E upregulation in *RNF43^{659mut}* CRCs. (A) Single-cell RNA sequencing analysis of epithelial cells in *RNF43^{wt}* and *RNF43^{659mut}* primary MSI-H CRCs (Cohort-1). Statistically significant genes included in our curated list of markers are shown. Statistical significance was determined using the Seurat (cutoffs: padj < 0.05 and $|\log_2\text{foldchange}| > 0.75$) R package. **(B)** Bulk RNA sequencing analysis of HT29 isogenic tumor cells. R1 to R3 in each category are biological replicates. Statistically significant genes included in our curated list of markers are shown. Statistical significance was determined using the Deseq2 (cutoffs: padj < 0.05) R package **(C)** Western blot (top) and quantitative analysis (bottom) of HLA-E expression in HT29 isogenic lines, HCT116 cells overexpressing *RNF43^{659mut}*, CRC PDOs, CRISPR-edited organoid line compared to controls. Statistical significance was determined using one-way ANOVA with Dunn's multiple comparisons test or Mann-Whitney test. Asterisks denote statistical significance (*P < 0.05).

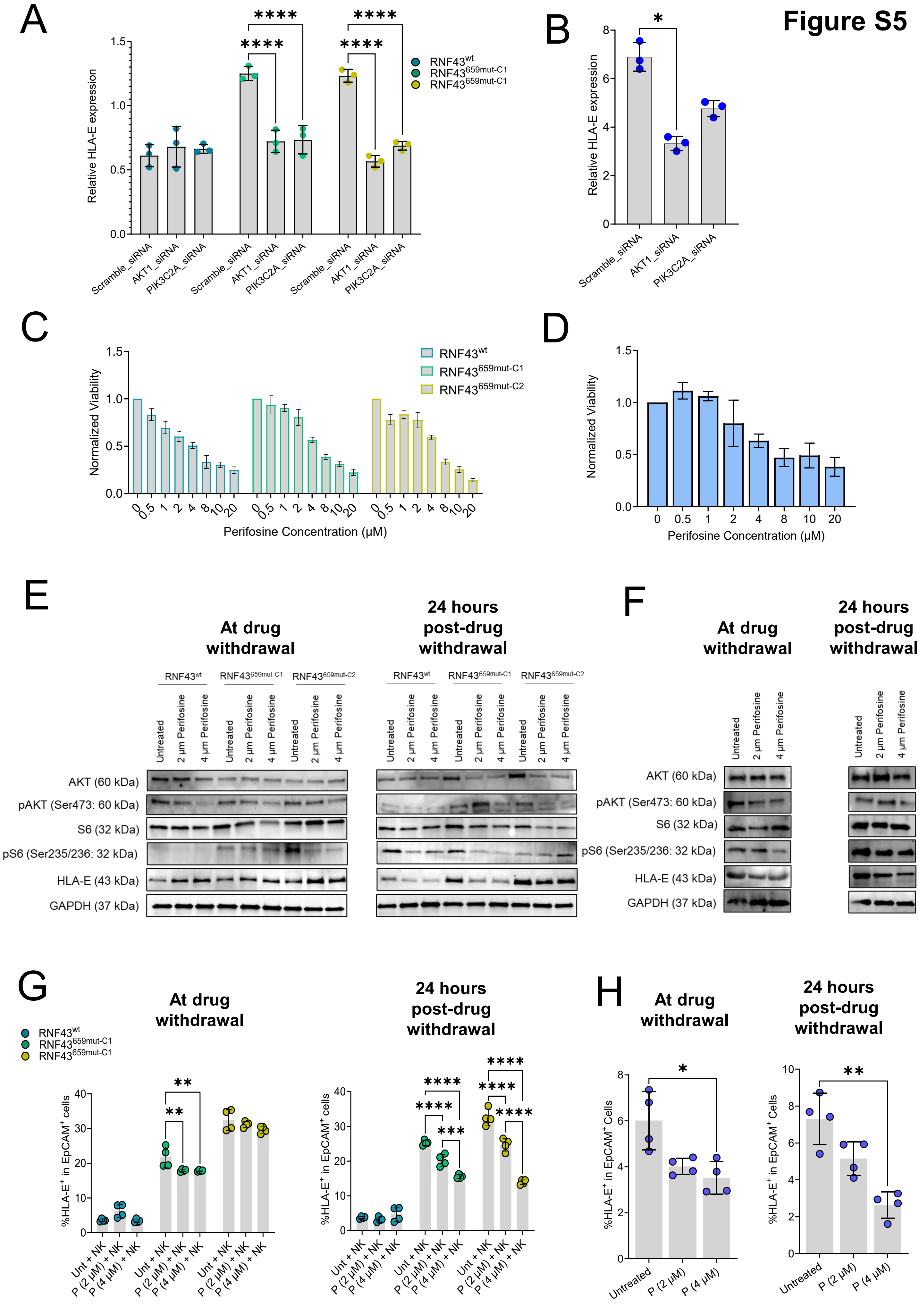

Figure S5
A
B
C
D
E
F
G
H
Relative HLA-E expression
Scramble_siRNA
AKT1_siRNA
PIK3C2A_siRNA
RNF43wt
RNF43659mut-C1
RNF43659mut-C1
Normalized Viability
Perifosine Concentration (µM)
RNF43wt
RNF43659mut-C1
RNF43659mut-C2
At drug withdrawal
24 hours post-drug withdrawal
RNF43wt
RNF43659mut-C1
RNF43659mut-C2
Untreated
2 µm Perifosine
4 µm Perifosine
AKT (60 kDa)
pAKT (Ser473: 60 kDa)
S6 (32 kDa)
pS6 (Ser235/236: 32 kDa)
HLA-E (43 kDa)
GAPDH (37 kDa)
RNF43wt
RNF43659mut-C1
RNF43659mut-C1
%HLA-E+ in EpCAM+ cells
Unt + NK
P (2 µM) + NK
P (4 µM) + NK
%HLA-E+ in EpCAM+ Cells
Untreated
P (2 µM)
P (4 µM)

Figure S5. Reduction of HLA-E Expression following PI3K/AKT Pathway Inhibition.
Quantification of immune blot to analyze HLA-E expression after siRNA mediated knockdown of *AKT1* and *PIK3C2A* in **(A)** *RNF43^{659mut}* HT29 isogenic tumor lines and **(B)** *RNF43^{659mut}* homozygous CRC PDOs. Perifosine dose response graphs for **(C)** *RNF43^{659mut}* HT29 isogenic tumor lines and **(D)** *RNF43^{659mut}* homozygous CRC PDOs analyzed after treatment for 72 hours. Following treatment with Perifosine (2 μM and 4 μM) for 4 hours, the drug was removed and PI3K/AKT pathway and HLA-E protein expression were assessed at the time of drug withdrawal (4 hours) and again at 28 hours after treatment initiation (24 hours after drug withdrawal) in **(E)** *RNF43^{659mut}* HT29 tumor lines and **(F)** Org3 (*RNF43^{659mut}* homozygous). Quantification of time-dependent reduction in HLA-E expression due to pharmacological inhibition of the PI3K/AKT pathway using Perifosine (2 μM and 4 μM) in **(G)** *RNF43^{659mut}* HT29 isogenic tumor lines and **(H)** RNF43^{659mut} homozygous CRC PDOs analyzed by flow cytometry. In **A-D** and **G, H**, data are presented as mean ± SD of pooled $\geq$3 independent biological experiments. Statistical significance was determined using two-way ANOVA for **A** and **G** or one-way ANOVA for **B** and **H** with Dunn's multiple comparisons test. Asterisks denote statistical significance (*P < 0.05; **P < 0.01; ***P < 0.001, ****P < 0.0001).

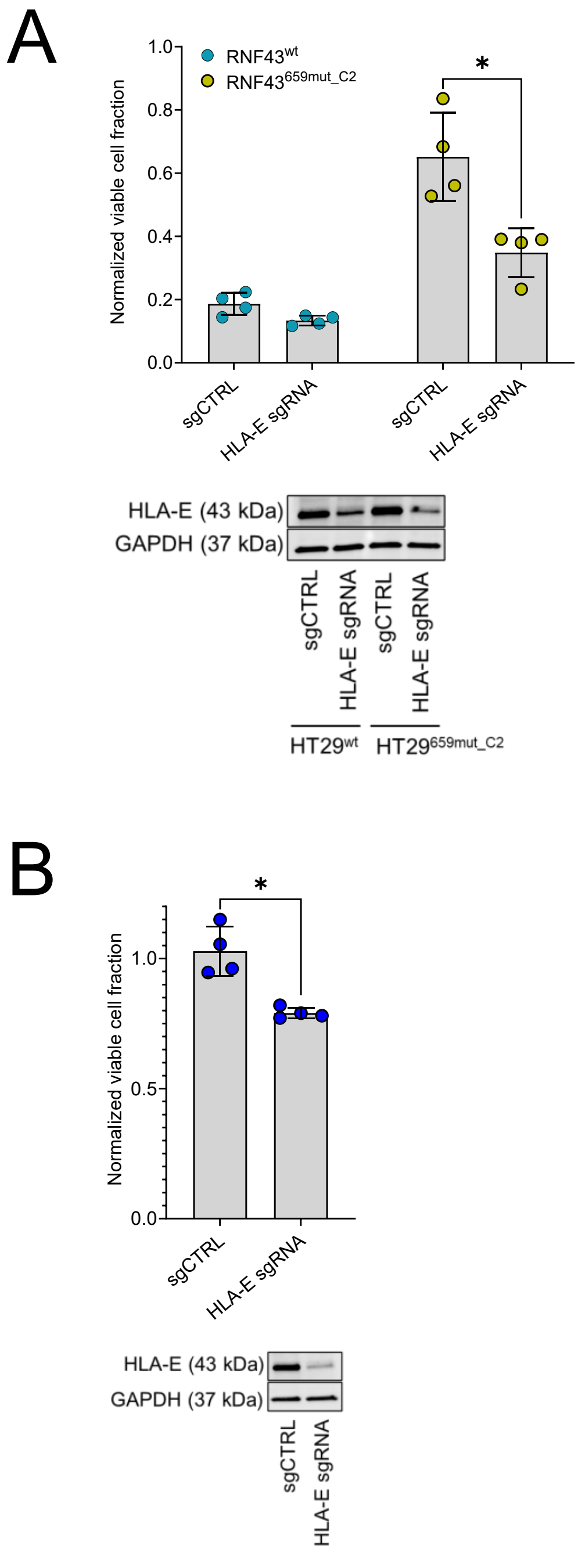

Figure S6. Generation and validation of HLA-E knockout RNF43659mut CRC models and assessment of NK cell cytotoxicity.

(A) NK cell cytotoxicity in co-culture with the *RNF43*^{wt} and *RNF43*$^{659mut\_C2}$ and isogenic line following CRISPR-mediated HLA-E knockout (top). Immunoblot confirming HLA-E knockout in the corresponding tumor cells (bottom). **(B)** NK cell cytotoxicity in co-culture with *RNF43*^{659mut} homozygous organoid 3 following CRISPR-mediated HLA-E knockout (top). Immunoblot validating HLA-E knockout in the corresponding organoid model (bottom). Cell viability was analyzed by flow cytometry. In graphs A and B, data are presented as mean ± SD of pooled 4 independent biological experiments. Statistical significance was determined using two-way ANOVA for A or Mann-Whitney test for B. Asterisks denote statistical significance (*$P < 0.05$).

Figure S7

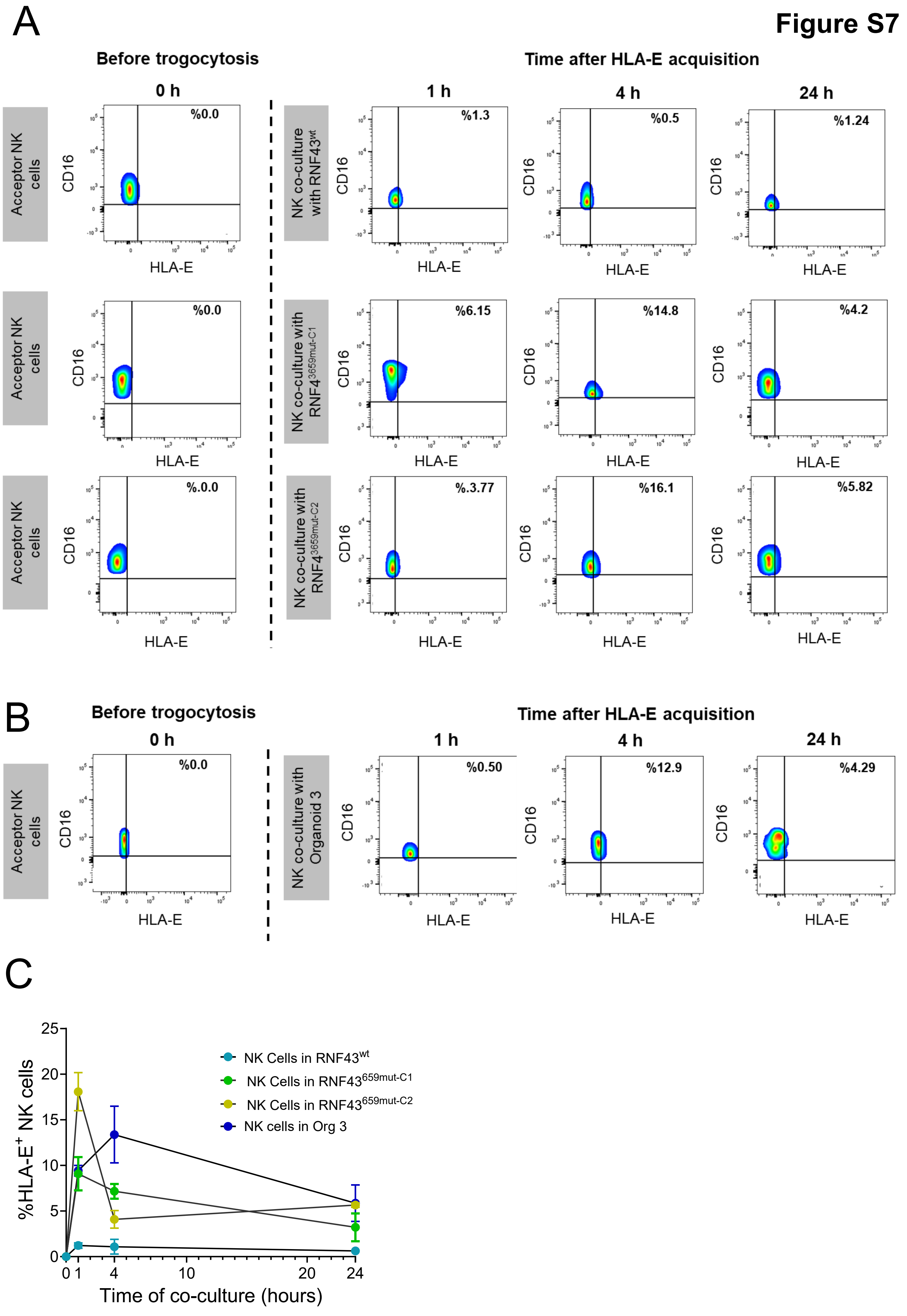

A
Before trogocytosis
Time after HLA-E acquisition
0 h
1 h
4 h
24 h
Acceptor NK cells
NK co-culture with RNF43wt
NK co-culture with RNF43659mut-C1
NK co-culture with RNF43659mut-C2
CD16
HLA-E
%0.0
%1.3
%0.5
%1.24
%0.0
%6.15
%14.8
%4.2
%.0.0
%.3.77
%16.1
%5.82
B
Before trogocytosis
Time after HLA-E acquisition
0 h
1 h
4 h
24 h
Acceptor NK cells
NK co-culture with Organoid 3
%0.0
%0.50
%12.9
%4.29
C
%HLA-E+ NK cells
Time of co-culture (hours)
NK Cells in RNF43wt
NK Cells in RNF43659mut-C1
NK Cells in RNF43659mut-C2
NK cells in Org 3

Figure S7. Flow cytometry analysis of HLA-E trogocytosis in RNF43^{659mut} CRC models. Representative flow cytometry plots showing transient HLA-E uptake by NK cells co-cultured with **(A)** *RNF43^{659mut}*, *RNF43^{wt}* HT29 isogenic lines or **(B)** Organoid 3. (**C)** Time-course assessment (0, 1, 4, and 24 hours) of HLA-E acquisition by NK cells co-cultured with HT29 isogenic lines and organoid 3. Quantification of HLA-E^{+} NK cells at each time point is shown. Data in **(C)** are presented as mean ± SD of pooled $\geq$3 independent biological experiments.

Figure S8

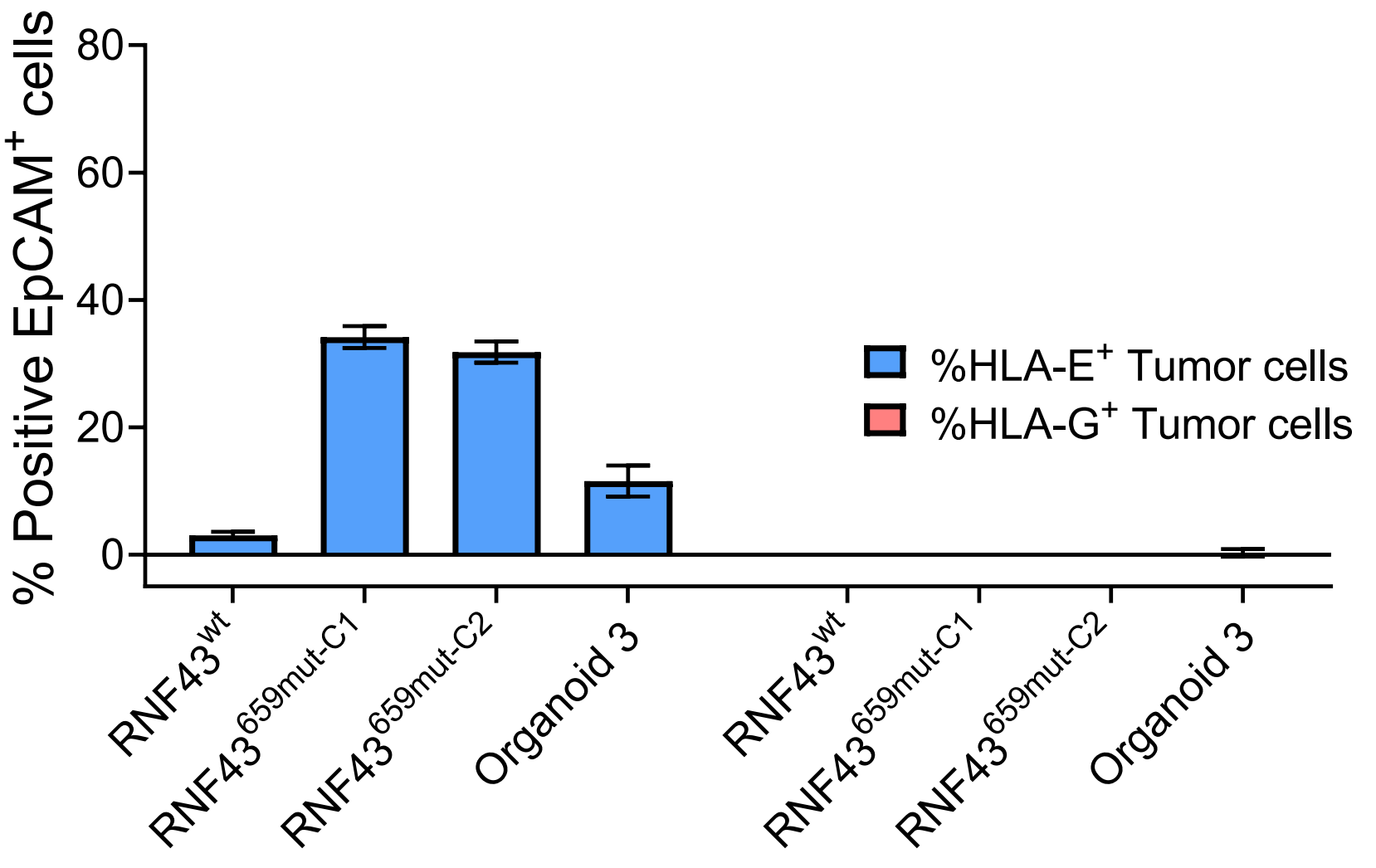

Figure S8. Flow cytometry analysis of HLA-E and HLA-G expression in tumor cells. HLA-E and HLA-G surface expression in HT29 isogenic tumor lines and Organoid 3 assessed by flow cytometry. Data are presented as mean ± SD of pooled 3 independent biological experiments.

Figure S9

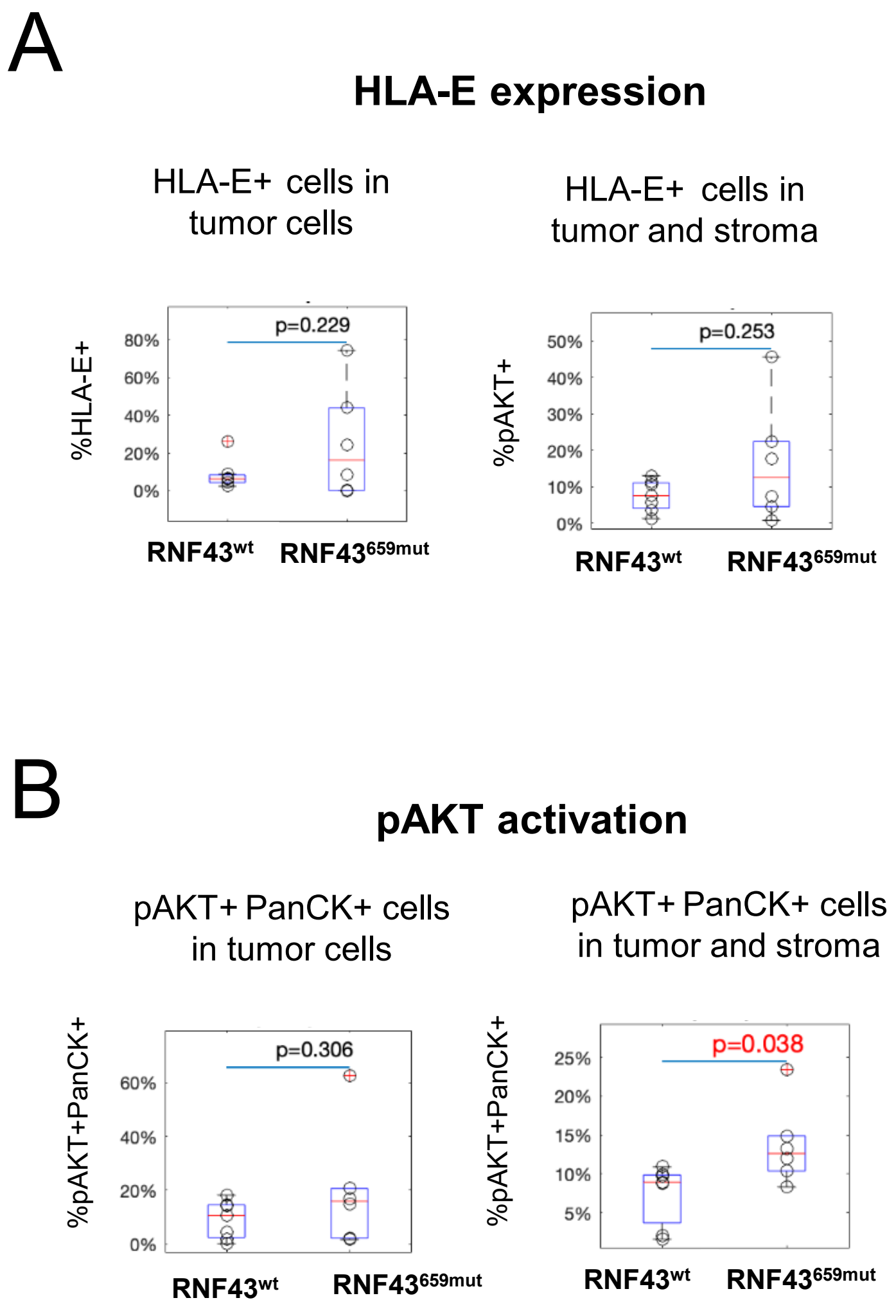

Figure S9. CyCIF spatial analysis of HLA-E expression and PI3K/AKT activation in MSI-H CRC. (A) HLA-E positive cells and **(B)** pAKT expressing cells in PanCK positive ($PanCK^+$) tumor boundaries, and across tumoral and stromal regions (all cells) from *$RNF43^{659mut}$* and *$RNF43^{wt}$* MSI-H CRC tumors. Statistical significance was determined using Welch's t-test for **A** and **B**. statistical significance ($P < 0.05$).

Supplementary Table 1. Cohort-3 Patient Clinical Data

No	Tumor ID	Sex	Site	Tissue collection procedure	Stage	MMR/MSI status	RNF43^{659mut} status
1	COL05396	F	Liver metastasis	Biopsy	IV	dMMR/MSI-h	Wild type
2	COL05575	M	Trasverse Colon	Surgery	IV	dMMR/MSI-h	Wild type
3	COL00501	M	Ascending Colon	Surgery	III	dMMR/MSI-h	Wild type
4	COL05031	F	Descending Colon	Surgery	IV	dMMR/MSI-h	Wild type
5	COL00504	M	Trasverse Colon	Biopsy	III	dMMR/MSI-h	Wild type
6	COL00608	M	Ascending Colon	Surgery	III	dMMR/MSI-h	Wild type
9	COL05254	F	Descending Colon	Surgery	IV	dMMR/MSI-h	Wild type
7	COL00612	F	Descending Colon	Surgery	IV	dMMR/MSI-h	Mutant
8	COL04915	M	Descending Colon	Surgery	III	dMMR/MSI-h	Mutant
10	COL04950	M	Ascending Colon	Biopsy	IV	dMMR/MSI-h	Mutant
11	COL05442	M	Descending Colon	Biopsy	IV	dMMR/MSI-h	Mutant
12	COL04956	M	Lymph node	Biopsy	IV	dMMR/MSI-h	Mutant
13	COL00466	M	Trasverse Colon	Surgery	IV	dMMR/MSI-h	Mutant
14	COL05064	F	Trasverse Colon	Surgery	IV	dMMR/MSI-h	Mutant

Supplementary Table 2. Clinical and Pathology Features of Patient-Derived Organoids

Organoid ID	Tissue type	Sex	Site	Stage	Mismatch Repair	Tissue collection procedure
Org 1	Tumor	Female	Right Colon/Hepatic Flexure	Stage II	Deficient	Surgery
Org 2	Tumor	Male	Right Colon/Ascedning Colon	Stage III	Deficient	Surgery
Org 3	Tumor	Female	Liver metastasis	Stage IV	Deficient	Surgery/Hepatectomy
Org 4	Tumor	Female	Right Colon	Stage III	Deficient	Mouse Xenograft
Normal	Normal	Female	Right Colon/Ascending Colon	NA	Intact	Surgery

Supplementary Table 3. RNF43 Status and Other Mutational Features of Patient-Derived Organoids

Organoid ID	Organoid type	$RNF43^{659mut}$ status	Nucleotide Change	Protein Change	WT reads	659fs reads	Total number of reads	VAF	MSIsensor2 Score	Mismatch Repair	Other mutations
**Org 1**	tumor	heterozygous	AC → A (deletion of C)	p.Gly659Valfs*41 ($RNF43^{659mut}$)	75	56	131	0.4275	89.89	Deficient	APC^{mut}, $KRAS^{wt}$, $PTEN^{wt}$, $BRAF^{wt}$, $MSH6^{mut}$
**Org 2**	tumor	heterozygous	AC → A (deletion of C)	p.Gly659Valfs*41 ($RNF43^{659mut}$)	81	79	160	0.4937	89.33	Deficient	APC^{mut}, $KRAS^{mut}$, $PTEN^{wt}$, $BRAF^{mut}$, $MSH6^{wt}$
**Org 3**	tumor	homozygous	AC → A (deletion of C)	p.Gly659Valfs*41 ($RNF43^{659mut}$)	0	150	150	1	87.61	Deficient	APC^{mut}, $KRAS^{wt}$, $PTEN^{wt}$, $BRAF^{wt}$, $MSH6^{wt}$, $PI3KCA^{wt}$
**Org 4**	tumor	homozygous	AC → A (deletion of C)	p.Gly659Valfs*41 ($RNF43^{659mut}$)	0	236	236	1	82.06	Deficient	APC^{wt}, $KRAS^{mut}$, $PTEN^{mut}$, $BRAF^{mt}$, $MSH6^{wt}$
**Normal**	normal	Wild-type	NA	NA	NA	NA	NA	NA	1.01	Intact	NA

Supplementary Table 4. Cohort-1 Patient Clinical Data and RNF43 Mutational Status of Tumors

Patient	Sex	Stage	RNF43^{659mut} status	MMR/MSI status	APC	KRAS	PTEN	BRAF	MSH6	PIK3CA	AKT1	AKT2	AKT3	MTOR	TP53	B2M
C106	M	IV	Mutant	dMMR/MSI-h	WT	WT	WT	Mutant	WT	WT	WT	WT	WT	WT	WT	WT
C109	F	III	Mutant	dMMR/MSI-h	Mutant	WT	WT	WT	WT	WT	WT	Mutant	WT	WT	WT	Mutant
C110	M	III	Mutant	dMMR/MSI-h	WT	WT	WT	Mutant	Mutant	WT	WT	WT	WT	WT	Mutant	WT
C111	F	III	Wild type	dMMR/MSI-h	WT	WT	WT	Mutant	WT	Mutant	WT	Mutant	WT	Mutant	WT	Mutant
C114	M	III	Wild type	dMMR/MSI-h	Mutant	WT	WT	Mutant	WT	WT	WT	WT	WT	WT	WT	Mutant
C115	M	III	Wild type	dMMR/MSI-h	WT	Mutant	Mutant	WT	Mutant	WT	WT	WT	WT	WT	WT	WT
C116	M	III	Wild type	dMMR/MSI-h	Mutant	WT	Mutant	WT	WT	WT	WT	WT	WT	WT	WT	WT
C118	M	III	Mutant	dMMR/MSI-h	WT	WT	WT	Mutant	WT	Mutant	WT	WT	WT	WT	WT	Mutant
C119	M	III	Mutant	dMMR/MSI-h	Mutant	Mutant	Mutant	WT	Mutant	WT	WT	WT	Mutant	WT	WT	Mutant
C122	F	III	Wild type	dMMR/MSI-h	Mutant	WT	WT	Mutant	Mutant	Mutant	WT	WT	WT	WT	WT	WT
C123	M	III	Mutant	dMMR/MSI-h	WT	WT	Mutant	Mutant	WT	WT	WT	WT	WT	WT	WT	WT
C130	F	IV	Mutant	dMMR/MSI-h	WT	WT	Mutant	Mutant	WT	WT	WT	WT	WT	WT	WT	WT
C132	M	IV	Wild type	dMMR/MSI-h	Mutant	Mutant	WT	WT	Mutant	WT	WT	WT	WT	WT	WT	WT
C137	F	III	Wild type	dMMR/MSI-h	Mutant	WT	WT	Mutant	WT	Mutant	WT	WT	WT	WT	WT	WT
C138	M	III	Mutant	dMMR/MSI-h	Mutant	Mutant	WT	WT	Mutant	WT	WT	WT	WT	Mutant	Mutant	Mutant
C139	F	IV	Mutant	dMMR/MSI-h	WT	WT	Mutant	WT	Mutant	Mutant	WT	WT	Mutant	WT	Mutant	Mutant
C142	F	III	Wild type	dMMR/MSI-h	Mutant	WT	WT	WT	Mutant	Mutant	WT	WT	WT	WT	Mutant	WT
C143	F	III	Wild type	dMMR/MSI-h	WT	WT	WT	WT	Mutant	Mutant	WT	WT	WT	Mutant	WT	Mutant
C144	M	III	Mutant	dMMR/MSI-h	WT	WT	WT	Mutant	Mutant	WT	WT	WT	Mutant	Mutant	WT	WT
C146	F	III	Mutant	dMMR/MSI-h	Mutant	WT	WT	Mutant	WT	Mutant	WT	WT	WT	WT	Mutant	WT
C147	F	IV	Wild type	dMMR/MSI-h	Mutant	WT	WT	Mutant	WT	WT	WT	WT	WT	WT	WT	WT
C151	F	III	Wild type	dMMR/MSI-h	WT	Mutant	Mutant	WT	WT	WT	WT	WT	WT	WT	WT	WT
C152	F	III	Mutant	dMMR/MSI-h	WT	WT	WT	Mutant	Mutant	Mutant	WT	Mutant	WT	Mutant	WT	WT
C154	F	II	Mutant	dMMR/MSI-h	Mutant	WT	WT	Mutant	WT	WT	WT	WT	WT	WT	WT	WT
C156	F	III	Mutant	dMMR/MSI-h	WT	WT	WT	Mutant	WT	Mutant	WT	WT	WT	WT	WT	WT
C158	F	III	Mutant	dMMR/MSI-h	WT	WT	WT	Mutant	WT	WT	WT	WT	WT	WT	WT	WT
C163	F	IV	Wild type	dMMR/MSI-h	Mutant	WT	WT	WT	WT	WT	WT	WT	WT	WT	Mutant	Mutant
C164	F	IV	Wild type	dMMR/MSI-h	WT	WT	WT	WT	Mutant	Mutant	WT	WT	WT	Mutant	Mutant	WT

C165	M	IV	Wild type	dMMR/MSI-h	WT	WT	WT	WT	Mutant	WT	Mutant	WT	WT	WT	Mutant	WT
C167	F	III	Wild type	dMMR/MSI-h	WT	WT	WT	WT	WT	WT	Mutant	WT	WT	WT	WT	Mutant
C168	M	II	Mutant	dMMR/MSI-h	WT	WT	Mutant	Mutant	Mutant	WT	WT	WT	WT	WT	Mutant	WT
C169	M	II	Wild type	dMMR/MSI-h	WT	WT	Mutant	WT	WT	WT	WT	WT	WT	WT	WT	WT
C170	F	III	Wild type	dMMR/MSI-h	Mutant	WT	WT	Mutant	Mutant	WT	WT	WT	WT	WT	WT	WT

Supplementary Table 5. List of Antibodies Used for CyCIF

Marker	Vendor	Cat#	Clone	Dilution
**Hoechst1**				
Donkey-anti-Goat	Invitrogen	A11055	Polyclonal	400
Donkey-anti-Rabbit	Invitrogen	A31572	Polyclonal	400
Donkey-anit-Mouse	Invitrogen	A31571	Polyclonal	400
**Hoechst2**				
CD56	R&D Systems	AF2408	Polyclonal	300
pAKT	Cell Signaling Technology	4060L	D9E	150
HLAE	Cell Signaling Technology	73269S	MEM-E/02	600
**Hoechst3**				
pS6_235	Cell Signaling Technology	4803S	D57.2.2E	300
panCK	Invitrogen	41-9003-82	AE1/AE3	600
CD45	Biolegned	304018	HI30	150
SMA	R&D Systems	IC1420S	1A4	300
**Hoechst4**				
CD4	Abcam	ab196372	EPR6855	500
CD3d	Abcam	ab208514	EPR4426	300
CD8a	Invitrogen	50-0008-82	AMC908	300
Ki67	R&D Systems	IC7617S	1297A	150
**Hoechst5**				
CD16	Santa Cruz Biotechnology	sc-20052	DJ130c	150
CDX2	Cell Signaling Technology	84638S	D11D10	300
NKG2A	Abcam	ab300745	EPR23737	300
MPO	Santa Cruz Biotechnology	sc-365436	A-5	150
**Hoechst6**				
TCF1	Cell Signaling Technology	6444S	C63D9	300
MLH1	Abcam	ab215303	EPR3894	300
CD103	Abcam	ab225153	EPR4166(2)	300
CK16	Thermofisher Scientific	606-510	LL025	300

Supplementary Table 6. List of Antibodies Used for Flow Cytometry and FACS				
**Immune marker**	**Fluorophore**	**Vendor**	**Cat#**	**Clone**
CD45	BV510	Biolegend	304036	HI30
CD45	Pacific blue	Biolegend	304029	HI30
CD3	PerCP/Cy5.5	Biolegend	317336	OKT3
CD56 (NCAM)	BV711	Biolegend	362542	5.1H11
CD56 (NCAM)	BV510	Biolegend	318340	HCD56
CD16	PerCP/Cy5.5	Biolegend	302028	3G8
CD314 (NKG2D)	FITC	Biolegend	320820	1D11
CD159a (NKG2A)	APC	Biolegend	375108	S19004C
CD158e1 (KIR3DL1, NKB1)	APC	Biolegend	312716	DX9
CD158a (KIR2DL1)	PE	Biolegend	374904	HP-DM1
CD366 (Tim3)	FITC	Biolegend	345022	F38-2E2
IFNɤ	BV605	Biolegend	506542	B27
TNFα	BV711	BD Biosciences	599421	MAb11
EpCAM (CD326)	BV421	Biolegend	324220	9C4
eBioscience™ Fixable Viability Dye eFluor™ 780	APC-Cy7	Invitrogen	65-0865-14	NA
HLA-E	APC	Biolegend	342606	3D12
HLA-G	PE	Biolegend	335906	87G
**Isotype controls**				
Mouse IgG1, κ	APC	Biolegend	400122	MOPC-21
Mouse IgG1, κ	BV605	Biolegend	400162	MOPC-21
Mouse IgG1, κ	BV711	Biolegend	400168	MOPC-21
Mouse IgG1, κ	FITC	Biolegend	400110	MOPC-21
Mouse IgG1, κ	PE	Biolegend	400114	MOPC-21
Mouse IgG1, κ	PerCP/Cy5.5	Biolegend	400150	MOPC-21

Supplementary Table 7. List of Antibodies Used for Immunoblotting			
**Immune marker**	**Vendor**	**Cat#**	**Clone**
AKT	Cell Signaling Technology	4685S	11E7
p-AKT (Ser473)	Cell Signaling Technology	9271S	Polyclonal
S6 Ribosomal Protein	Cell Signaling Technology	2217S	5G10
p-S6 Ribosomal Protein (Ser235/236)	Cell Signaling Technology	4858S	D57.2.2E
HLA-E	Cell Signaling Technology	73269S	MEM-E/02
GAPDH	Cell Signaling Technology	2118S	14C10
ECL Mouse IgG, HRP-linked whole Ab (from sheep)	Amersham	NA931-1ML	Polyclonal
ECL Rabbit IgG, HRP-linked whole Ab (from donkey)	Amersham	NA934-1ML	Polyclonal
